# Microscopical and elemental FESEM and Phenom ProX-SEM-EDS analysis of osteocyte- and blood vessel-like microstructures obtained from fossil vertebrates of the Eocene Messel Pit, Germany

**DOI:** 10.7717/peerj.1618

**Published:** 2016-01-21

**Authors:** Edwin Cadena

**Affiliations:** Paleoherpetology, Senckenberg Research Institute, Frankfurt am Main, Germany

**Keywords:** Osteocytes, Blood vessels, Eocene, Germany, Messel Pit, Turtles, Crocodiles, Mammals, Molecular paleontology

## Abstract

The Eocene (∾48 Ma) Messel Pit in Germany is a UNESCO World Heritage Site because of its exceptionally preserved fossils, including vertebrates, invertebrates, and plants. Messel fossil vertebrates are typically characterized by their articulated state, and in some cases the skin, hair, feathers, scales and stomach contents are also preserved. Despite the exceptional macroscopic preservation of Messel fossil vertebrates, the microstructural aspect of these fossils has been poorly explored. In particular, soft tissue structures such as hair or feathers have not been chemically analyzed, nor have bone microstructures. I report here the preservation and recovery of osteocyte-like and blood vessel-like microstructures from the bone of Messel Pit specimens, including the turtles *Allaeochelys crassesculpta* and *Neochelys franzeni*, the crocodile *Diplocynodon darwini*, and the pangolin *Eomanis krebsi*. I used a Field Emission Scanning Electron Microscope (FESEM) and a Phenom ProX desktop scanning electron microscope (LOT-QuantumDesign) equipped with a thermionic CeB6 source and a high sensitivity multi-mode backscatter electron (BSE) for microscopical and elemental characterization of these bone microstructures. Osteocyte-like and blood vessel-like microstructures are constituted by a thin layer (∾50 nm thickness), external and internal mottled texture with slightly marked striations. Circular to linear marks are common on the external surface of the osteocyte-like microstructures and are interpreted as microbial troughs. Iron (Fe) is the most abundant element found in the osteocyte-like and blood vessel-like microstructures, but not in the bone matrix or collagen fibril-like microstructures. The occurrence of well-preserved soft-tissue elements (at least their physical form) establishes a promising background for future studies on preservation of biomolecules (proteins or DNA) in Messel Pit fossils.

## Introduction

Osteocytes are the most abundant cells in the bone tissue of all vertebrates (Bonewald, 2011, references therein). Together with blood vessels and mineralized collagen fibrils, osteocytes have been reported and described from different fossil sites, geologic ages, and different lineages of vertebrates (Enlow & Brown, 1956; Pawlicki, 1978; Pawlicki, 1985; Pawlicki & Nowogrodzka-Zagorska, 1998; Schweitzer et al., 2005; Schweitzer, Wittmeyer & Horner, 2007a; Schweitzer et al., 2009; Cadena & Schweitzer, 2012; Cadena & Schweitzer, 2014). Characterization and description of the fossil soft-tissue bone microstructures (osteocytes, blood vessels and collagen fibrils, abbreviated here as OBvF-like) have been accomplished using different optical tools, including bone thin sections observed under transmitted and polarized light microscopes, isolation of OBvF-like microstructures by removal of the mineral phase with acid and their subsequent analysis using scanning, transmission, and thermal field emission electron microscopy (SEM, TEM, and FESEM respectively) (see Schweitzer et al., 2008; Cadena & Schweitzer, 2012; Schweitzer et al., 2013). OBvF-like microstructures have also been characterized chemically using mass spectrometry and immunological tools, principally to show evidence not only of their molecular composition, but also of their function as capsules for preservation of original organic components, particularly proteins and potentially DNA material (Schweitzer et al., 2007b; Schweitzer et al., 2013; Cleland et al., 2015).

The Messel Pit, a globally famous fossil site recognized by UNESCO as a World Natural Heritage Site because of the exceptional preservation as well as the diversity of its fossils, which include vertebrates, invertebrates, and plants (see i.e., Wedmann, Bradler & Rust, 2007; Smith, 2009; Vinther et al., 2009; Müller et al., 2011 references therein), is located near the city of Darmstadt in Germany. The vertebrates are preserved in articulation, often with associated originally “soft” tissues such as hair, scales or feathers, and even occasionally in situ stomach contents. The age of Messel Pit is early-middle Eocene (∾48 Ma) (Lenz et al., 2015 and references therein). Despite the exceptional macroscopic preservation of Messel Pit fossil vertebrates, the microstructural preservation of these fossils has been poorly explored. Only two studies described potential bacteria and other microorganisms in bone fragments, scales and eyes of fish, as well as in coprolites using TEM (Wuttke, 1983; Liebig, Westall & Schmitz, 1996). Whether this preservation could extend to the micro- or nano-scales is currently unknown.

Here I report the microscopic preservation of OBvF-like microstructures from three different lineages of vertebrates, including two species of turtles: the pleurodire or side-necked *Neochelys franzeni* (Schleich, 1993), recently redescribed by Cadena (2015), and the cryptodire or hidden-necked turtle *Allaeochelys crassesculpta* (Harrassowitz, 1922), commonly found in pairs that died while copulating (Joyce et al., 2012). As well as, one crocodile taxon, *Diplocynodon darwini*, and one mammal, the pangolin *Eomanis krebsi* (Storch & Martin, 1994). I present a first attempt to determine their elemental composition using Phenom ProX-SEM-EDS. Finally, I discussed the paleobiological and molecular implications of OBvF-like microstructural preservation in vertebrates from Messel Pit.

## Materials and Methods

### Fossil samples

**Figure 1:**
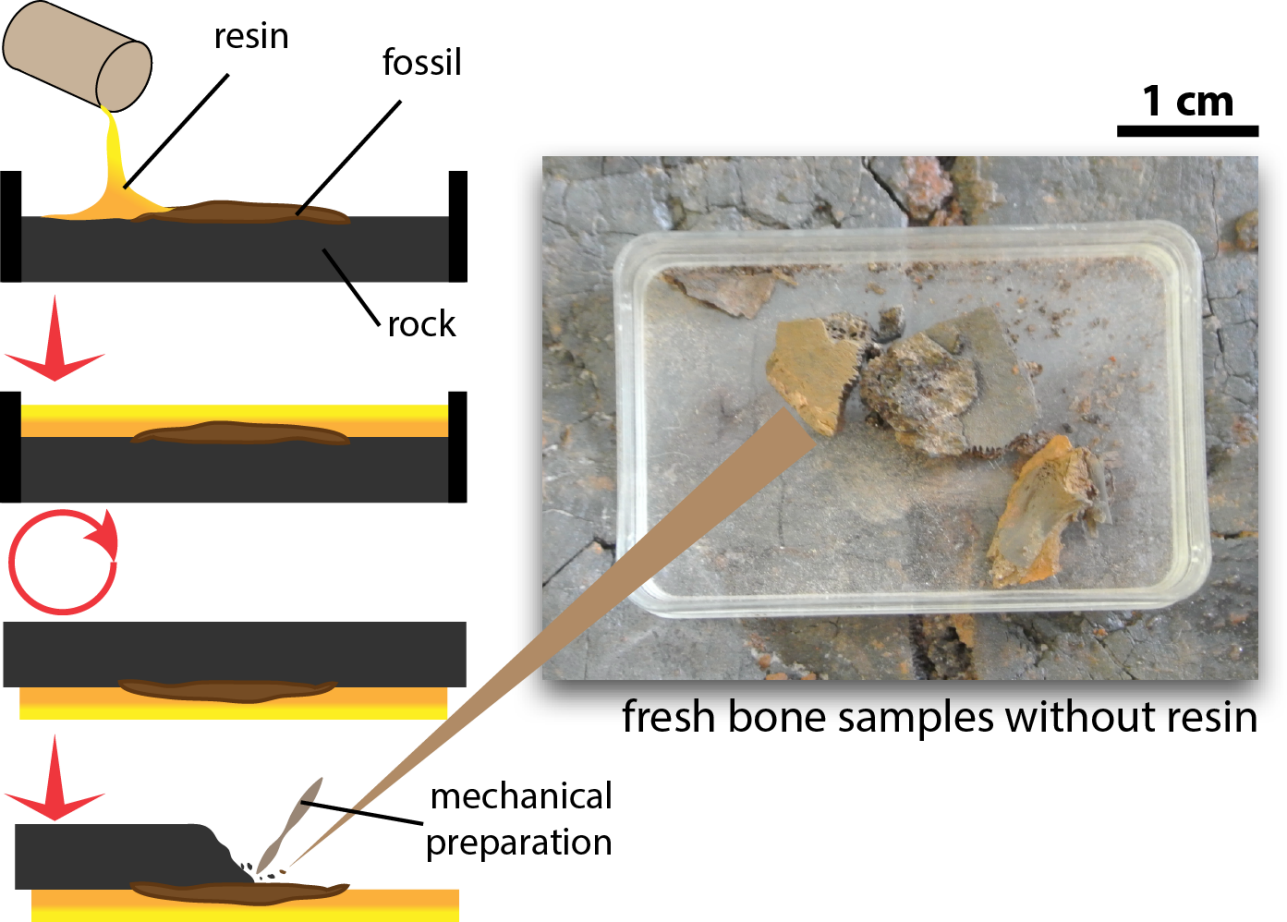
Procedure of excavation and preparation of Messel Pit turtles and the obtention of the bone samples analyzed in this study.

Tiny fragments of bone (≤1 cm^3^ from the fossil specimens, which were left after mechanical preparation of the exposed surface and preserved without application of any resin, glue, or stabilizing additives, were sampled to explore preservation of OBvF-like microstructures (Fig. 1). All samples belong to specimens housed at the Messel vertebrate collection of the Senckenberg Naturmuseum (SMF ME) in Frankfurt am Main, Germany. A total of nine samples were taken, including three turtle specimens: *Allaeochelys crassesculpta* SMF ME 2449, *Neochelys franzeni* SMF ME 1091, *Euroemys kehreri* SMF ME 1807; one mammal: *Eomanis krebsi* SMF ME 1573; one amphibian: undetermined frog bone SMF ME 11432; one crocodile: *Diplocynodon darwini* SMF ME 898; one lizard: osteoderms of *Eurheloderma* sp.; one bird? *Messelornis* SMF ME uncatalogued specimen; and one fish: *Atractosteus* sp. SMF ME 2751. The taxonomic attribution of the specimens was taken from the Senckenberg Museum collections database, some of the samples belong to specimens published as followed: *Al. crassesculpta* SMF ME 2449 (Joyce et al., 2012), *N. franzeni* SMF ME 1091 (Cadena, 2015), and *E. krebsi* SMF ME 1573 (Storch & Martin, 1994).

### OBvF-like microstructure isolation and transmitted light microscopy

Bone fragments were demineralized using isodium ethylenediaminetetraacetic acid (EDTA) (0.5 M, pH 8.0 filter-sterilized using a 0.22 µm filter) as previously described (Schweitzer et al., 2008) for a period of 5 days to 2 weeks, or until OBvF-like microstructures emerged. These OBvF-like microstructures were observed using a Zeiss Axioskop 2 plus biological-transmitted light microscope, 40x, and 100X (oil immersion lens) and imaged with a Nikon camera coupled to the microscope.

### FESEM-BSE microscopy

OBvF-like microstructures emerging from bone after demineralization were collected into 1.5 ml microcentrifuge tubes. Samples were rinsed 3 times (at 1,000 rpm) in E-pure water to remove EDTA buffers. After centrifugation, samples were passed over a 0.4 micron Nylon cell strainer, then the strainer containing fossil remains (thin-small bone matrix remains and the OBvF-like microstructures) was mounted on individual SEM stubs, and affixed to the stub with silver polish. Samples were imaged using a Field Emission Scanning Electron Microscope (FESEM Sigma VP SEM, Carl Zeiss NTS) located at the Geosciences Department, Goethe Universität, Frankfurt, Germany. Samples were imaged without coating under 0.90 kV EHT (primary-beam energy) with a working distance of 3.0 mm. Elemental analyses were conducted using a Phenom ProX desktop scanning electron microscope (LOT-QuantumDesign) equipped with a thermionic CeB6 source and a high sensitivity multi-mode backscatter electron (BSE) detector, 0.15 kV EHT (primary-beam energy), also at the Geosciences Department, Goethe Universität, Frankfurt, Germany. At least 3 different points were analyzed for elemental composition for each sample studied.

## Results

### OBvF-like microstructure recovery

Of the nine samples of different vertebrate groups (turtles, mammals, birds, crocodiles, lizards, amphibians, and fish) studied here, only four preserved abundant OBvF-like microstructures: two of the turtles (*Allaeochelys crassesculpta* and *Neochelys franzeni*), the crocodile and the mammal.

### Morphological characterization of OBvF-like microstructures from Messel Pit

**Figure 2:**
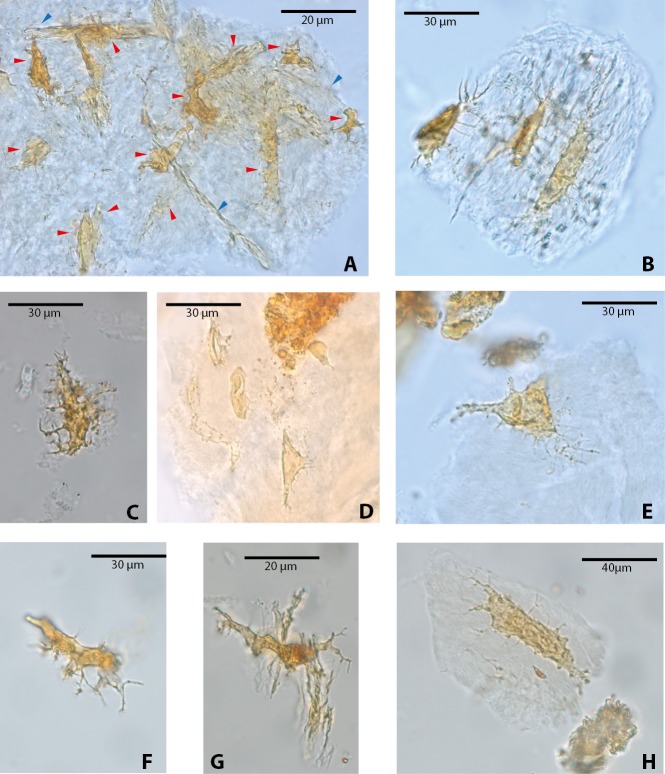
Osteocytes-like microstructures from fossil turtles from Messel Pit, under transmitted light microscopy. (A–E) osteocytes-like microstructures from *Allaeochelys crassesculpta*. Red arrows (osteocytes-like microstructure), blue arrows (collagen fibrils-like microstructures) in (A). (F–H) osteocytes-like microstructures from *Neochelys franzeni*. Some of the osteocytes-like and collagen fibrils-like microstructures are still embedded in a remaining thin layer or bone, transparent to lighter colored material.

**Figure 3:**
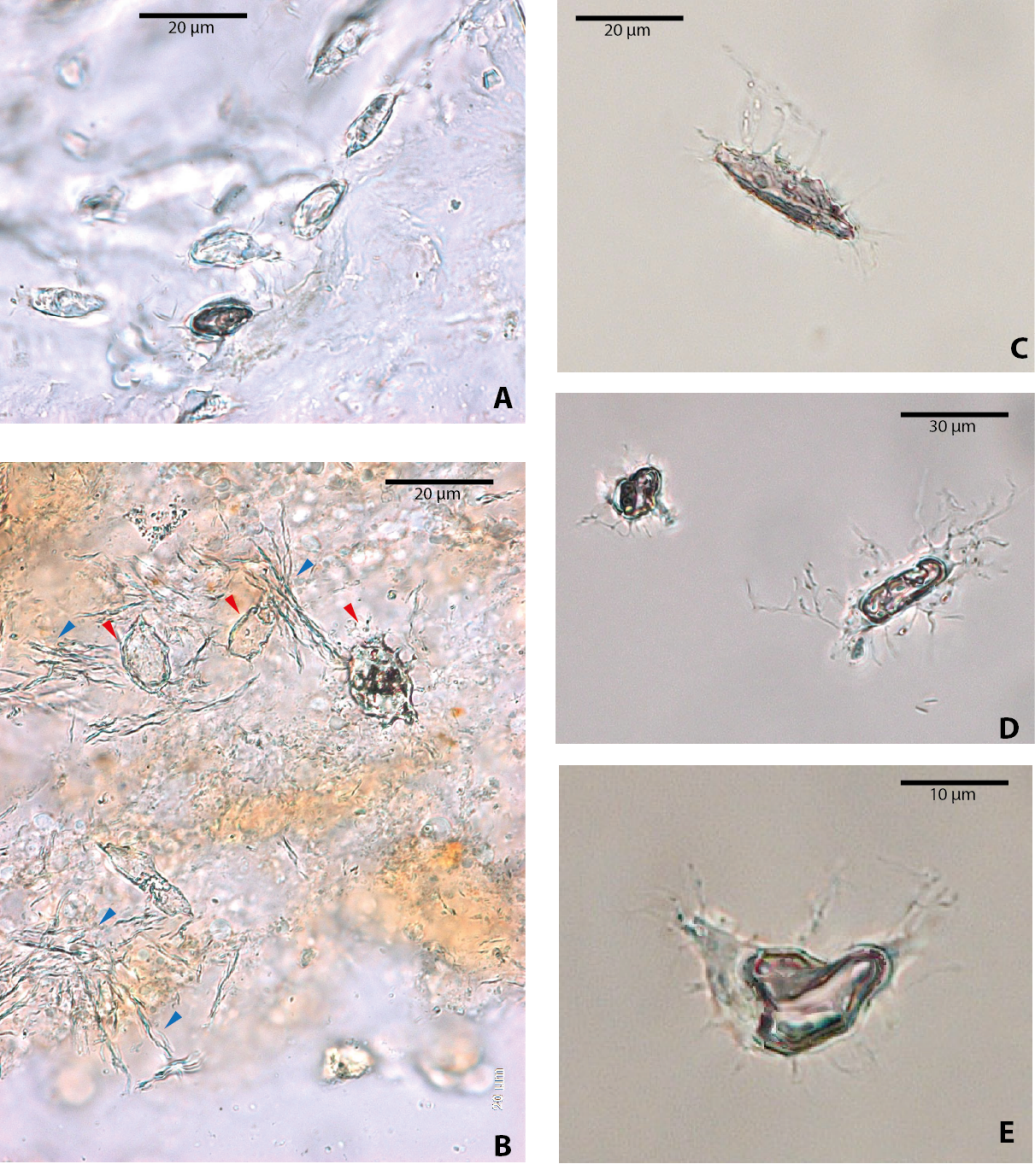
Osteocytes-like microstructures from a fossil crocodile and a fossil mammal from Messel Pit, under transmitted light microscopy. (A–B) osteocytes-like microstructures from the crocodile, *Diplocynodon* sp. Red arrows (osteocytes-like microstructures), blue arrows (collagen fibrils-like microstructures). (C–E) osteocytes-like microstructures from the pangolin *Eomanis krebsi.*

Among vertebrate cells, osteocytes present a unique morphology because during development they become entrapped in the matrix they secrete; to obtain nutrients and eliminate waste, their cell membranes form extensions known as filopodia (Bonewald, 2011; Lin & Xu, 2011). The morphology of the osteocytes and the bony cavities in which they are housed in life, known as osteocyte lacunae, have been previously described (Schweitzer et al., 2005; Schweitzer, Wittmeyer & Horner, 2007a; Schweitzer et al., 2009; Schweitzer et al., 2013; Cao et al., 2011; Cadena & Schweitzer, 2012, and references therein). These same morphological features are exhibited by the osteocytes-like of vertebrates from Messel Pit described herein. Osteocytes-like microstructures from the turtles *Allaeochelys crassesculpta* and *Neochelys franzeni* vary from semi-oval to elongated (between 20 and 40 µm) (Figs. 2A–2E), to very elongated (∾50 µm) (Figs. 2F–2H), almost all of them preserving second- and third-level ramifications of filopodia, and are yellow to orange in color. In contrast to turtles, the osteocytes-like microstructures from the crocodile *Diplocynodon darwini* (Figs. 3A–3B) and the mammal *Eomanis krebsi* (Figs. 3C–3E) are slightly smaller, particularly the semi-oval osteocyte-like microstructures (between 10 and 25 µm). Additionally, they are translucent, and no staining is evident. However, they also exhibit second- and third-level ramifications of filopodia. Broken or fragmented osteocyte-like microstructures were also commonly observed (Figs. 3D–3E).

**Figure 4:**
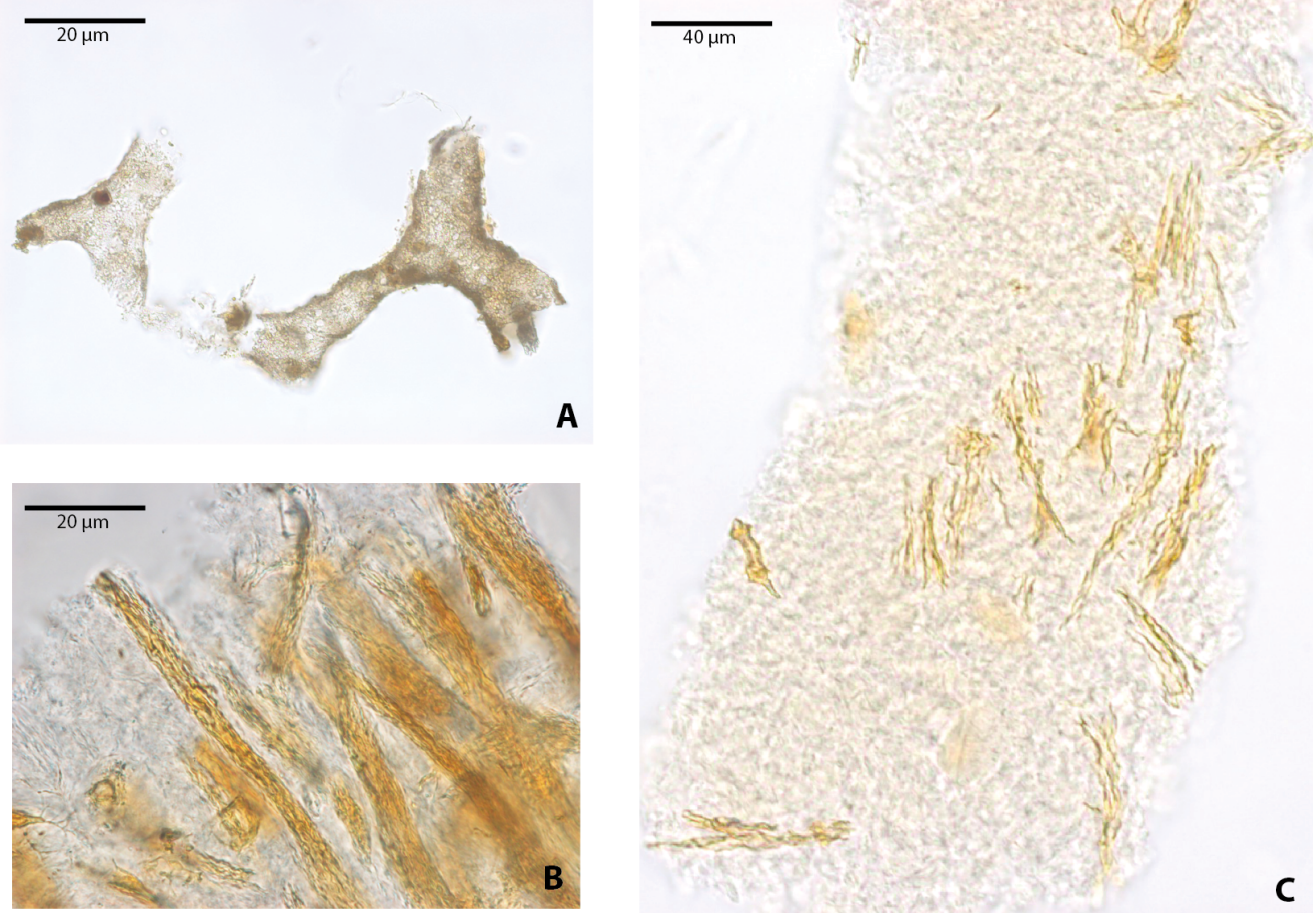
Blood vessels-like and collagen fibrils-like microstructures from *Allaeochelys crassesculpta* under transmitted light microscopy. (A) blood vessels-like microstructures. (B–C) collagen fibrils-like microstructures isolated or in bundles. Red arrows (osteocytes-like microstructures), blue arrows (collagen fibrils-like microstructures). Collagen fibrils-like microstructures are still embedded in a remaining thin layer or bone, transparent to lighter colored material.

Blood vessel-like and collagen fibril-like microstructures were also recovered after demineralization of bone, particularly in *Allaeochelys crassesculpta* (Fig. 4). The blood vessel-like microstructures are characterized by a thin wall with a microgranular texture (Fig. 4A), similar in both texture and diameter (∾10 µm average) to the blood vessel-like microstructures reported previously from a Paleocene bothremydid turtle from Colombia (Cadena & Schweitzer, 2014). Collagen fibril-like microstructures were also found forming stripes or groups of several fibrils (Figs. 4B–4C). The fibrils appear stained, exhibiting yellow to orange coloration, yet retained the characteristic plywood-like arrangement exhibited in almost all vertebrate bone, including human bone (see Varga et al., 2013). The collagen fibril-like microstructures were texturally distinct from both the blood vessels-like and the osteocytes-like recovered from the same bone samples. It is important to point out here that at present the definition of these blood vessel- and collagen fibril-like microstructures is based on their resemblance to the morphology of the same microstructures in extant turtles (see Cadena & Schweitzer, 2012).

**Figure 5:**
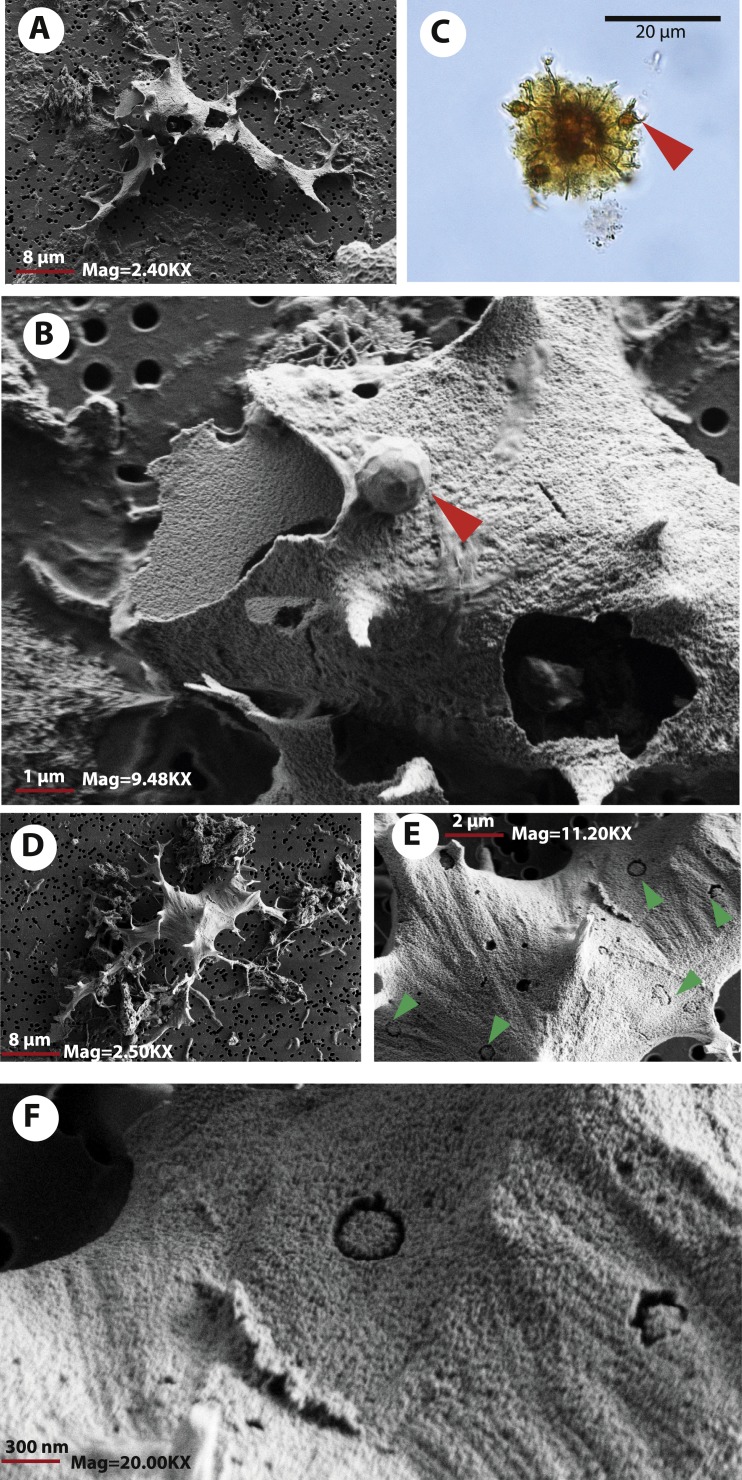
Osteocytes-like microstructures from Messel Pit turtles under FESEM. (A–B) osteocyte-like microstructures from *Neochelys franzeni*, showing breaking edges and a sphere with polygons on the exterior surface (red arrow), potentially of siderite or goethite. (C) osteocyte-like microstructure from *N. franzeni* under transmitted light microscopy, with spheres at the filopodia (red arrow). (D–F) osteocyte-like microstructure of *Allaeochelys crassesculpta* with circular and linear to irregular marks or troughs (green arrows in (E)).

FESEM analyses of the osteocyte-like microstructures from Messel, particularly for the two species of turtles, *Neochelys franzeni* (Figs. 5A–5C) and *Allaeochelys crassesculpta* (Figs. 5D–5F), show that they consist of a material exhibiting a mottled texture externally and internally (Fig. 5B) (see elemental characterization section, for elemental characterization of this material). Some of the osteocyte-like microstructures have in their filopodia or sometimes over the exterior surface spheres that under transmitted light microscopy exhibit the same coloration as the osteocyte-like microstructures (Fig. 5C), and under FESEM they are not only spherical, but also have a pentagonal net-like divisions (Fig. 5B). The elemental composition of these spheres is still unknown at this time, since none of those was recovered during the analysis with the Phenom ProX-SEM-EDS. Another notable feature of the external surface of these osteocyte-like microstructures is the occurrence of marks or troughs, most of them circular, others linear to irregular (Figs. 5E–5F). Linear branching troughs have been reported to occur in vascular canals attributed to microbial activity (Kaye, Gaugler & Sawlowicz, 2008). The external surface of some of the osteocyte-like microstructures also exhibits weak striations (Figs. 5E–5F).

As it was observed under transmitted light microscopy, some of the osteocyte-like microstructures show breakage, some being cleaved in two (Fig. 3E). Using FESEM the break edges were examined, shedding light on the physical properties of the material constituting these osteocyte-like microstructures. For example the osteocyte shown in Figs. 5A–5B, suffered breakage during the filtering-mounting processes, possibly due to desiccation, preserving its upper left portion next to the main body of the microstructures. This suggests a fragile rather than a ductile material, with clearly defined, relatively straight-uniform edges, without evidence of bending (flexible) or fibrous components, which is also the case of other broken regions of the osteocyte-like microstructures (see Fig. 5B, right-down region). A 3-dimensional visualization “in situ” of the internal bone microstructural elements of the Messel Pit vertebrates wait to be done using nano-computer tomography, something that could provide not only more information about their original morphology, but also about their intrabone variation, as well as their volumetric density, distribution, and network connectivity.

### Phenom ProX-SEM-EDS analysis of OBvF-like microstructures from Messel Pit

**Figure 6:**
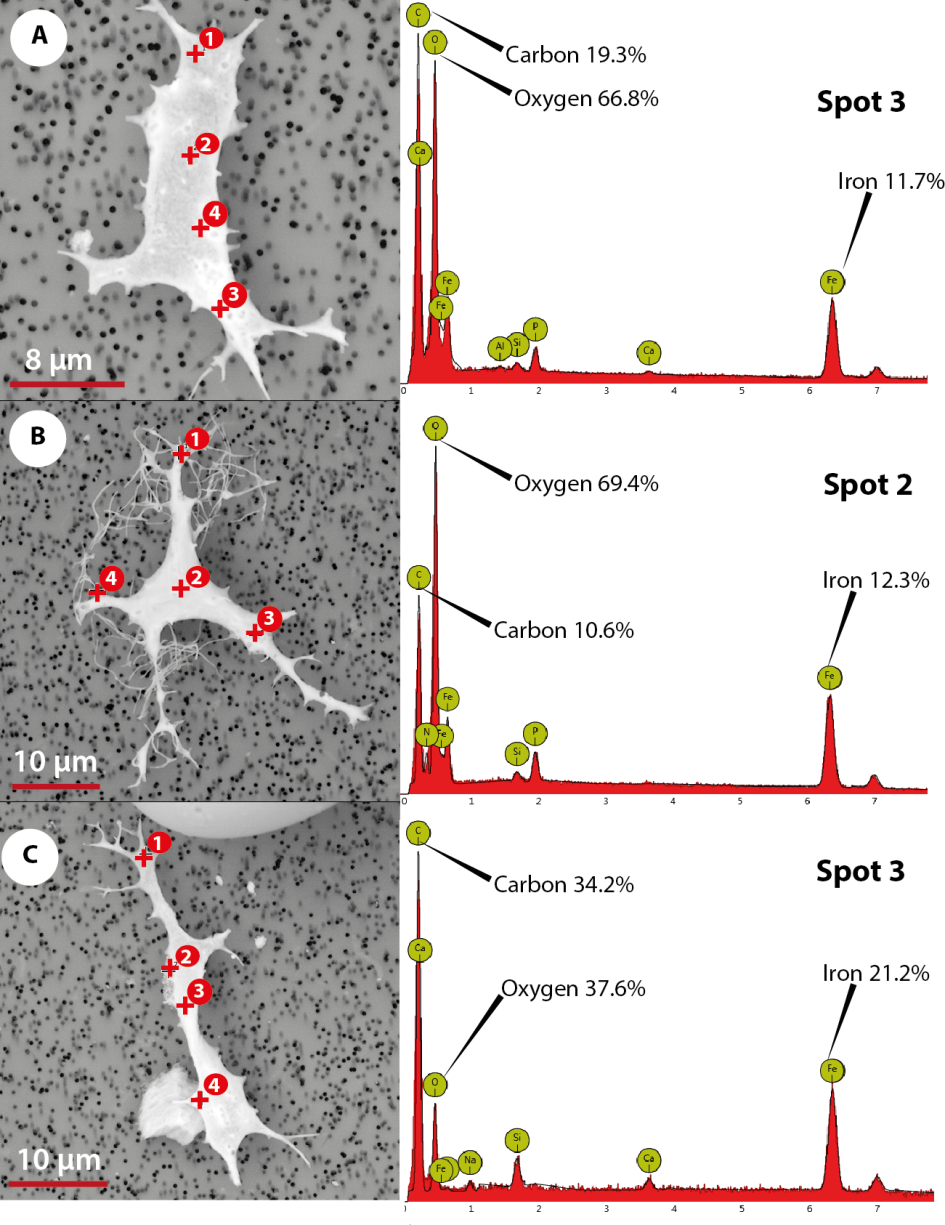
Elemental composition of osteocytes-like microstructures from Messel Pit turtles under Phenom ProX-SEM-EDS. (A–B) osteocytes-like microstructure of *Allaeochelys crassesculpta*, on the right spectrum obtained from the elemental X-ray spectroscopy analysis indicating the spot in the osteocyte-like where the reading was obtained. (C) osteocyte-like microstructure of *Neochelys franzeni.*

**Figure 7:**
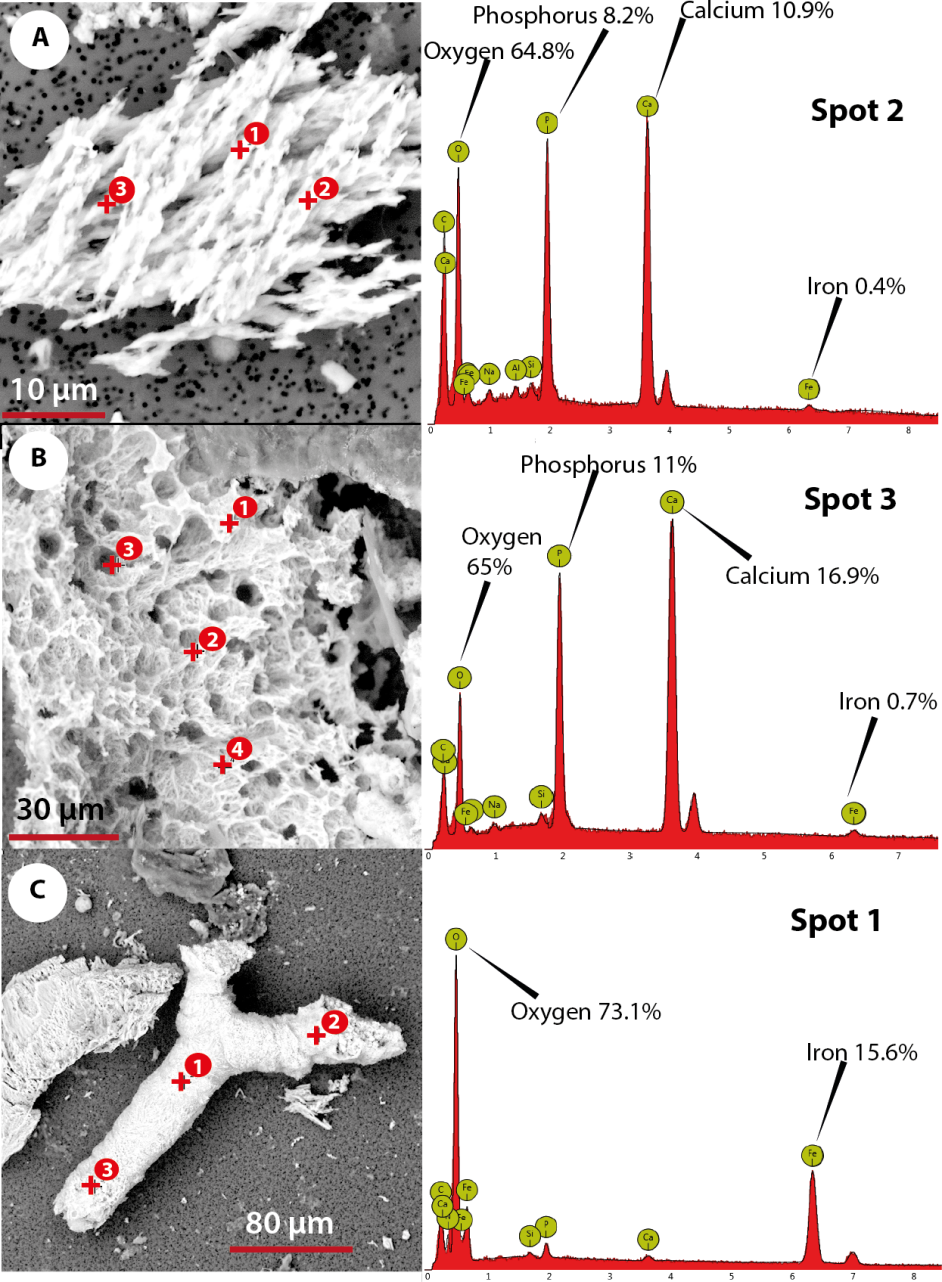
Elemental composition of collagen fibrils-like, bone matrix, blood vessel-like microstructures from Messel Pit turtles, under Phenom ProX-SEM-EDS. (A) collagen fibrils-like microstructures of *Allaeochelys crassesculpta*, on the right column, the spectrum obtained from the elemental X-ray spectroscopy analysis indicating the spot in the collagen fibril-like microstructures where the reading was obtained. (B) bone matrix remain of *Allaeochelys crassesculpta*, with the respective elemental composition spectrum on the right. (C) blood vessel-like microstructure of *Neochelys franzeni*, with the respective elemental composition spectrum on the right.

The elemental analysis of OBvF-like microstructures in the two turtle species using FESEM-BSE is summarized in Figs. 6–7. These included three osteocyte-like microstructures for *Allaeochelys crassesculpta*, two of them shown in Figs. 6A–6B (complete results in Supplemental Information 1), and three osteocyte-like microstructures for *Neochelys franzeni*, one shown in Fig. 6C (complete results in Supplemental Information 2). All osteocyte-like microstructures from both species show occurrence of three major elements: oxygen (O), carbon (C) and iron (Fe), in all cases with values above 10%; other elements like phosphorus (P), calcium (Ca), nitrogen (N) and silicon (Si) are also present but in minimal percentages usually below 5% (see Supplemental Informations 1 and 2). In contrast to the elemental composition of osteocyte-like microstructures, the collagen fibril-like microstructures and the remains of the bone matrix lack a high concentration of Fe (less than 1%). Instead, the composition is dominated by O, Ca, and P (all these with percentages above 8%) (Figs. 7A–7B, and Supplemental Informations 1 and 2). Blood vessel-like microstructures, however, like the osteocytes are also rich in Fe with values about 15% (Fig. 7C).

## Discussion

The occurrence of OBvF-like microstructures in vertebrates from the Messel Pit, particularly in turtles (*Allaeochelys crassesculpta* and *Neochelys franzeni*) but also from the pangolin *Eomanis krebsi* and the crocodile *Diplocynodon darwini*, confirms that the exceptional preservation of these fossils extends from the macroscopical (whole skeletons, some with hair or skin impressions) down to the microstructural level inside the bone tissue. These data support the idea that such preservation is surprisingly common in bone from deep time (Schweitzer, Wittmeyer & Horner, 2007a; Bertazzo et al., 2015). Here, I show that this observation, previously described in turtles of Jurassic, Cretaceous and Cenozoic age (Cadena & Schweitzer, 2012), applies as well to the Messel Pit: a fossil locality with a distinct depositional environment (volcanic maar lake), age (early-middle Eocene ∾48 Ma), and geographical location (central Germany). This observation is in agreement with the global, pan-environmental, and taxon-independent pattern preservation of OBvF-like microstructures reported previously (Schweitzer, Wittmeyer & Horner, 2007a; Cadena & Schweitzer, 2012; Cadena & Schweitzer, 2014)

The lack of recovery of OBvF-like microstructures from the rest of the samples does not necessarily mean that they should be ignored or excluded from future studies, because there is also a chance that more intensive sampling of these or other specimens of the same taxa will turn out positive for the occurrence of OBvF-like microstructures. This could be explained by intrabone preservation variation, potentially conditioned by changes in microenvironmental conditions and interactions between bone and rock matrix.

I hypothesize here that the linear branching troughs observed in some of the osteocytes-like microstructures attributed to microbial activity potentially occurred posterior to the stabilization of the osteocytes-like in the state that is preserved in the bone, but more sampling is required of osteocytes-like under FESEM and even experiments with living osteocytes exposed to microbes in order to test it.

The breaking of some of the osteocytes-like microstructures suggests a fragile rather than a ductile material, with clearly defined, relatively straight-uniform edges, without evidence of bending (flexible) or fibrous components, which is also the case of other broken regions of the osteocyte-like microstructures (see Fig. 5B, right-down region). A 3-dimensional visualization “in situ” of the internal bone microstructural elements of the Messel Pit vertebrates wait to be done using nano-computer tomography, something that could provide not only more information about their original morphology, but also about their intrabone variation, as well as their volumetric density, distribution, and network connectivity.

The abundant occurrence of iron (Fe) in the osteocyte- and blood vessel-like microstructures from *A. crassesculpta* and *N. franzeni*, but not in their collagen fibril-like microstructures and bone matrix remains, indicates a potential mechanism of chemical sequestration between microstructures, and also compositional differences between these microstructures would not be expected if all were the result of common diagenetic processes. According to data shown by Schweitzer et al. (2014), iron contributes to preservation of soft tissue and molecules in deep time by a possible combination of two mechanisms: free-radical-mediated crosslinking of proteins and cell membrane lipids resulting in a natural fixation, and hemoglobin-related anti-microbial activity. These mechanisms could also have operated in Messel Pit vertebrates, thus favored by the posited anoxic bottom water and abundance of iron-rich minerals in Messel oil shale rocks (including siderite and messelite; Tütken, 2014). Characterization of the mineral form of the iron found in the osteocyte- and blood vessel-like microstructures from Messel is unknown at this point, and it will have to be analyzed by future studies.

It must be emphasized that the elemental composition of osteocyte- and blood vessel-like microstructures reported here did not show high levels of nitrogen, an element that would be expected if any original proteins or even DNA were preserved in these microstructures. Furthermore I consider that the absence of nitrogen is not related to masking by carbon as mentioned can occur in some EDX detectors (Schweitzer et al., 2008), because the instrument use a CeB6 electron source, which according to the specification of the equipment generates the highest number of X-rays in its market segment, minimizing the possible masking of elements effect. Additionally, the occurrence of similar percentage of iron in transparent and yellow to orange stained osteocytes-like suggests that elements other than iron could be responsible for the variation of color of these OBvF-like microstructures.

## Conclusions

Here, I have described and provided microscopic and preliminary compositional data on OBvF-like microstructures recovered from Messel vertebrate remains. Although any additional molecular-chemical or immunological characterization of these microstructures is beyond the scope of this study. The data presented here encourage that such studies should be part of future research on Messel Pit vertebrates, including for example mass spectrometry and sequencing of potential protein remains, Phenom ProX-SEM-EDS with elemental mapping, X-ray photoelectric spectroscopy to test, for example, the mineral form of iron, as mentioned in Schweitzer et al. (2008), as well as iron-related protein antibodies. All these tools will further test whether original microstructures are in fact preserved, as well as contribute in the formulation and understanding of the mechanisms of preservation. Because osteocyte-like microstructures are preserved for more than one group of vertebrates from this depositional setting (turtles, mammals, and crocodiles), and because for some groups, including primates (Franzen et al., 2009) and horses (Franzen, 2006), the Messel Pit fossils represent some of the most basal forms of these groups, molecular data have potential to shed light on evolutionary relationships, as well as the mode and tempo of evolution at the molecular level.

## Supplemental Information

10.7717/peerj.1618/supp-1 Supplemental Information 1 Elemental analyses of OBvF-like microstructures of *Allaeochelys crassesculpta* SMF ME 2449 conducted using a Phenom ProX desktop scanning electron microscope (LOT-QuantumDesign) equipped with a thermionic CeB6 source and a high sensitivity multi-mode backscatter electron (BSE) detector, 0.15 kV EHT (primary-beam energy), also at the Geosciences Department, Goethe Universität, Frankfurt, Germany

10.7717/peerj.1618/supp-2 Supplemental Information 2 Elemental analyses of OBvF-like microstructures of *Neochelys franzeni* SMF ME 1091 conducted using a Phenom ProX desktop scanning electron microscope (LOT-QuantumDesign) equipped with a thermionic CeB6 source and a high sensitivity multi-mode backscatter electron (BSE) detector, 0.15 kV EHT (primary-beam energy), also at the Geosciences Department, Goethe Universität, Frankfurt, Germany

## Additional Information and Declarations

### Data Availability

The following information was supplied regarding data availability: The research in this article did not generate any raw data
