# Supplementary material for: Microscopical and elemental FESEM and Phenom ProX-SEM-EDS analysis of osteocyte- and blood vessel-like microstructures obtained from fossil vertebrates of the Eocene Messel Pit, Germany
Source: PeerJ. 2016 Jan 21;4:e1618. doi: 10.7717/peerj.1618 (PMC4727973; doi:10.7717/peerj.1618)
Supplement: Supplemental Information 1 [file peerj-04-1618-s001.pdf]

Supplementary information 1

Elemental analyses of OBvF-like microstructures of *Allaeochelys crassesculpta* SMF ME 2449 conducted using a Phenom ProX desktop scanning electron microscope (LOT-QuantumDesign) equipped with a thermionic CeB6 source and a high sensitivity multi-mode backscatter electron (BSE) detector, 0.15 kV EHT (primary-beam energy), also at the Geosciences Department, Goethe Universität, Frankfurt, Germany.

"Osteocyte-like microstructure" 01 (2 spots marked with cross and number)

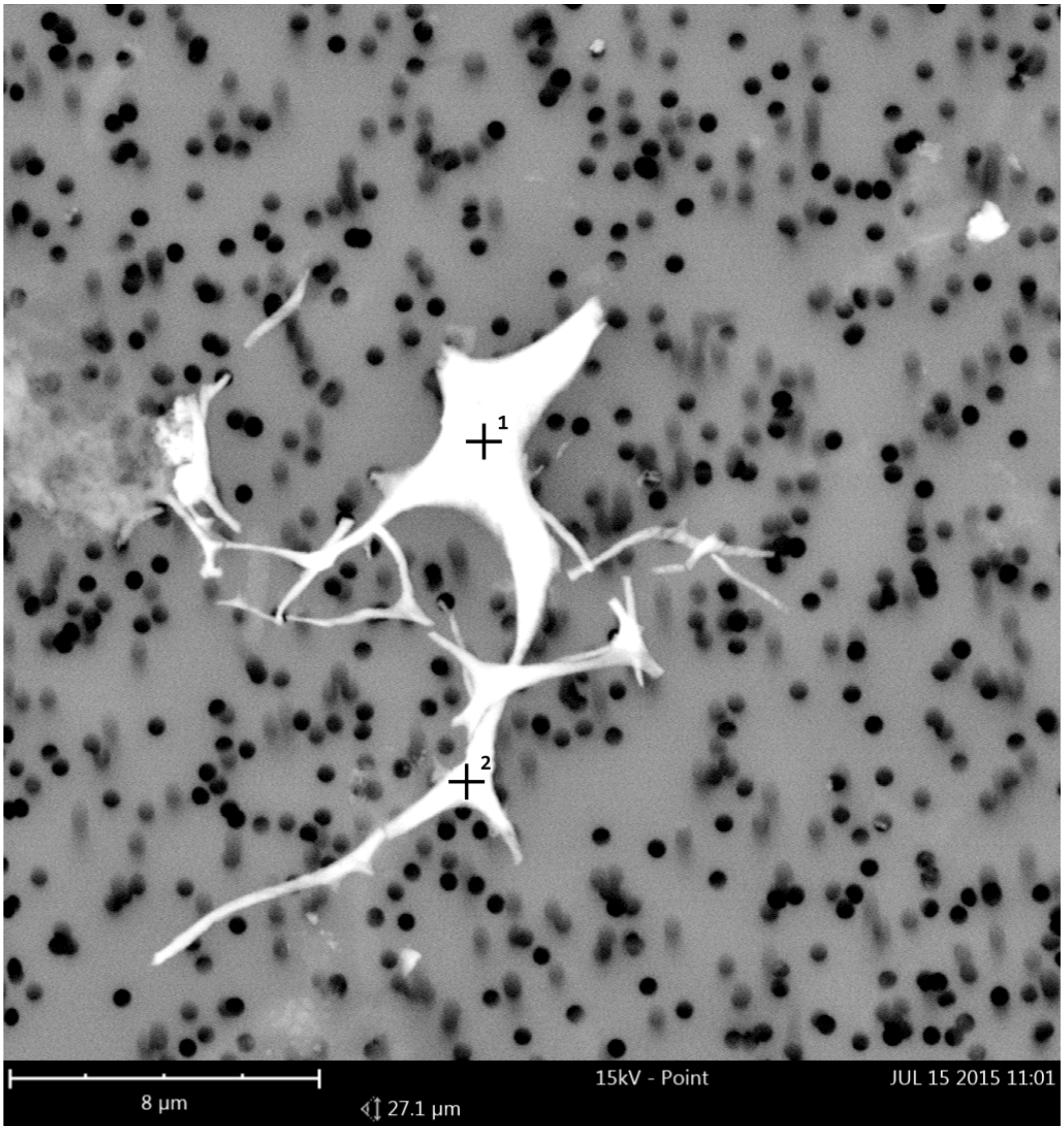

1. spot

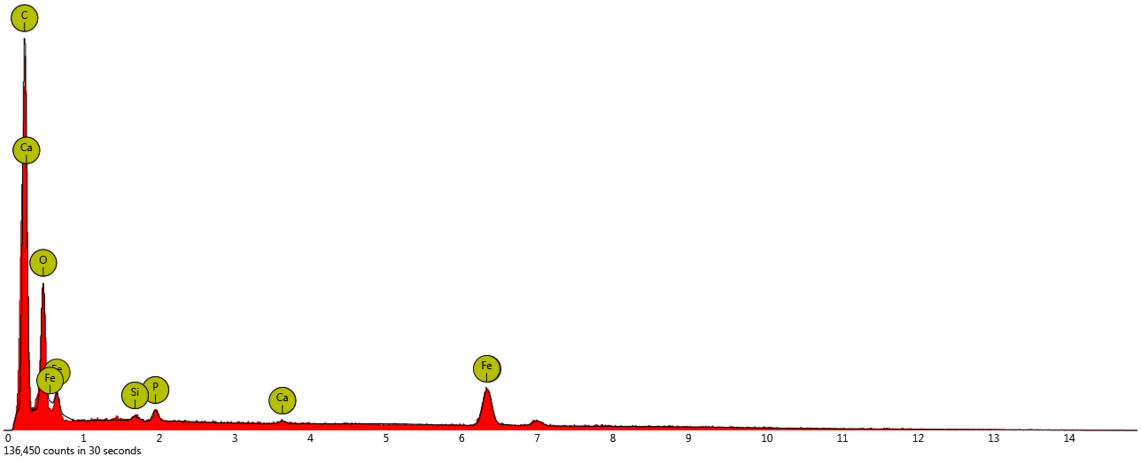

Disabled elements: B

Element Number	Element Symbol	Element Name	Atomic Concentration	Error
6	C	Carbon	33.1	1.1
8	O	Oxygen	56.7	0.0
26	Fe	Iron	8.5	0.1
15	P	Phosphorus	1.0	0.3
14	Si	Silicon	0.4	0.5
20	Ca	Calcium	0.3	0.7

2. spot

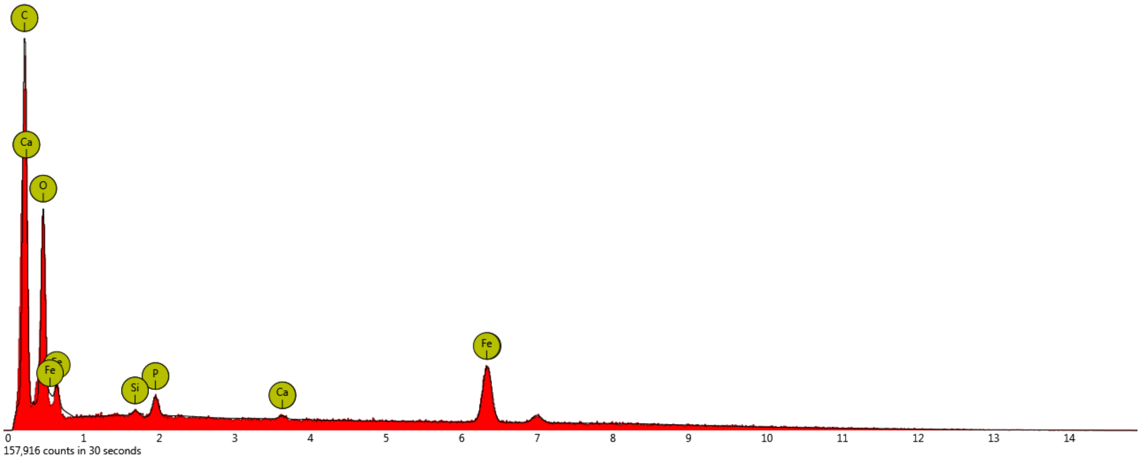

Disabled elements: B

Element Number	Element Symbol	Element Name	Atomic Concentration	Error
6	C	Carbon	26.9	1.4
8	O	Oxygen	61.0	0.0
26	Fe	Iron	10.2	0.1
15	P	Phosphorus	1.4	0.3
20	Ca	Calcium	0.3	0.8

14	Si	Silicon	0.3	0.6

“Osteocyte-like microstructure” 02 (4 spots marked with cross and number)

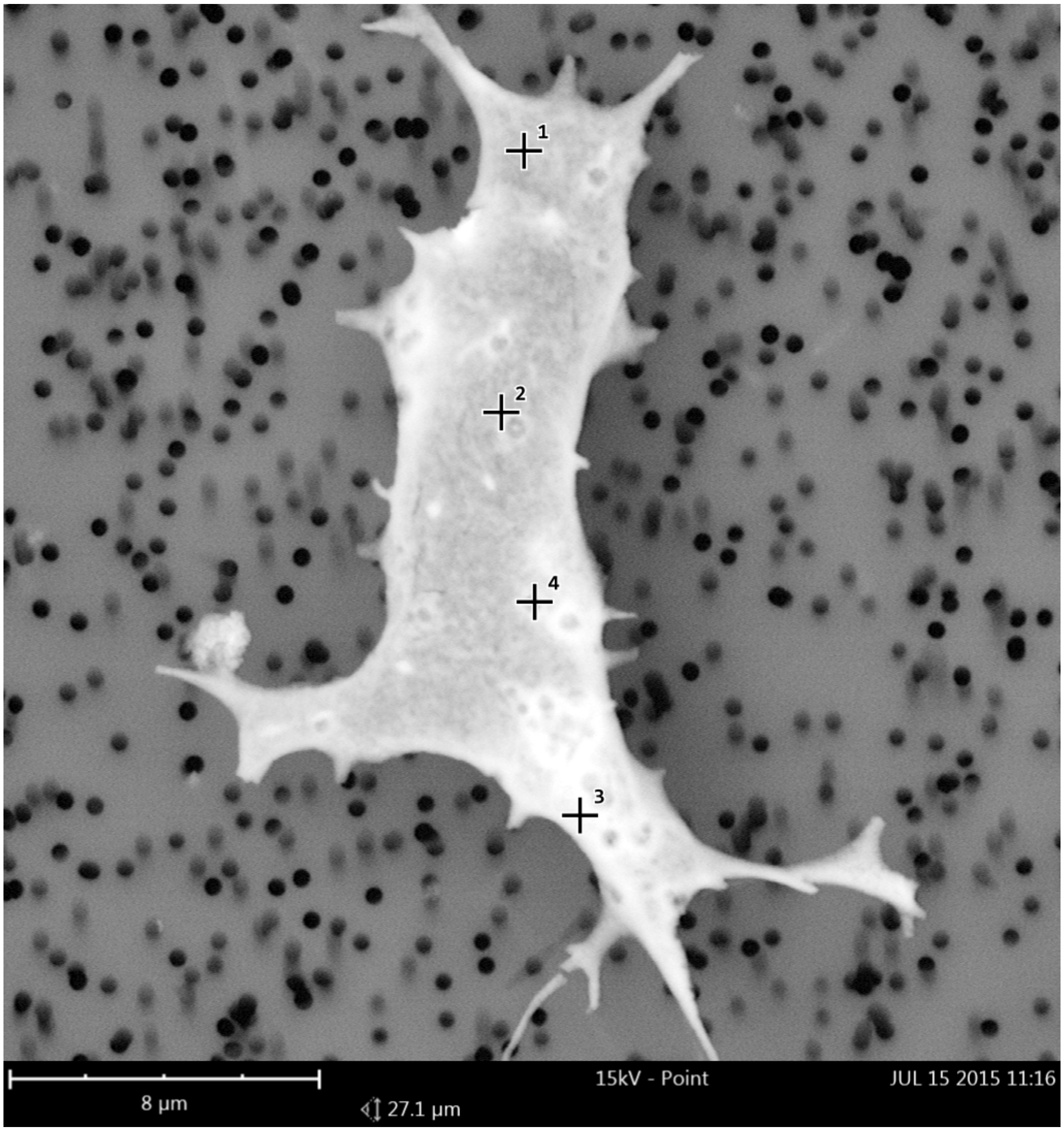

1. spot

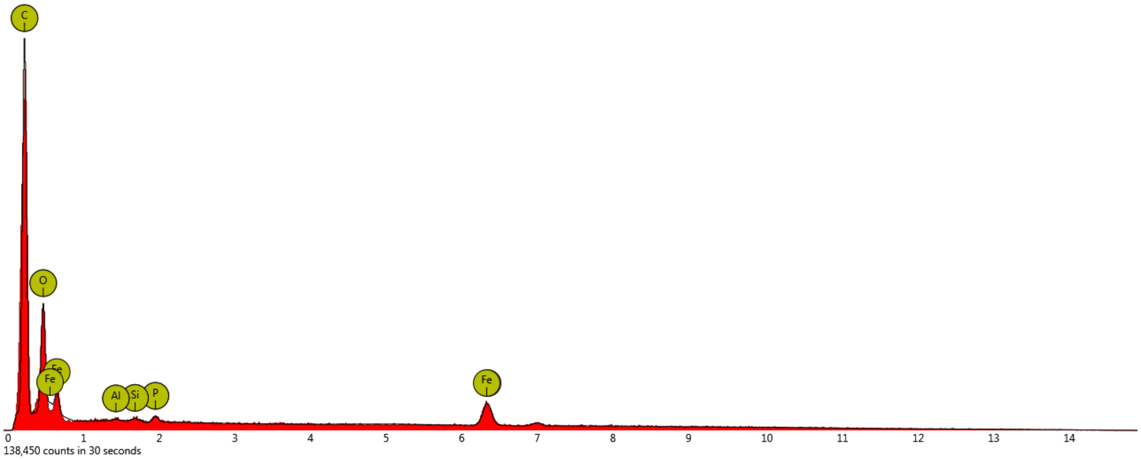

Disabled elements: B

Element Number	Element Symbol	Element Name	Atomic Concentration	Error
6	C	Carbon	35.1	1.7
8	O	Oxygen	57.7	0.0
26	Fe	Iron	6.2	0.1
15	P	Phosphorus	0.6	0.5
14	Si	Silicon	0.2	0.8
13	Al	Aluminium	0.2	0.6

2. spot

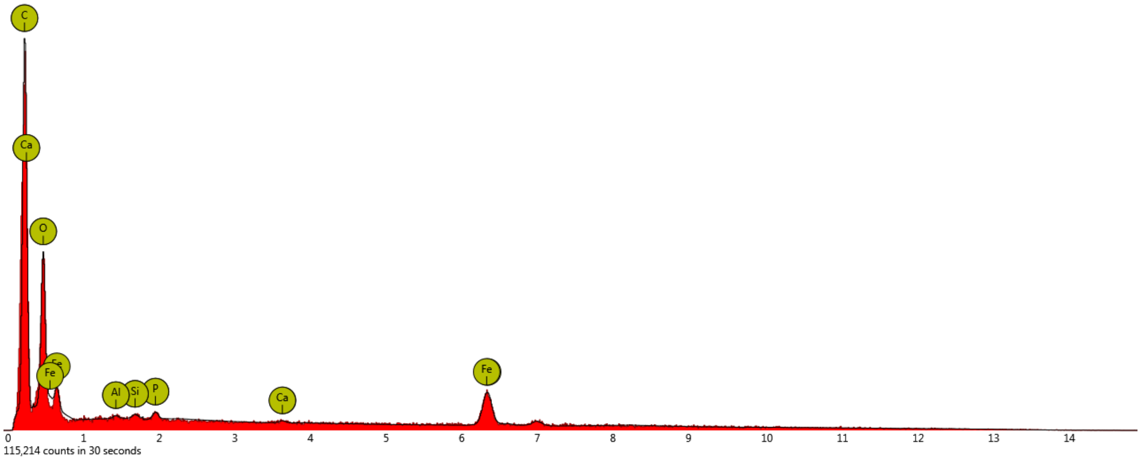

Disabled elements: B

Element Number	Element Symbol	Element Name	Atomic Concentration	Error
6	C	Carbon	30.1	1.1
8	O	Oxygen	61.2	0.0
26	Fe	Iron	7.2	0.1

15	P	Phosphorus	0.6	0.7
14	Si	Silicon	0.3	1.0
13	Al	Aluminium	0.3	0.7
20	Ca	Calcium	0.2	0.9

3. spot

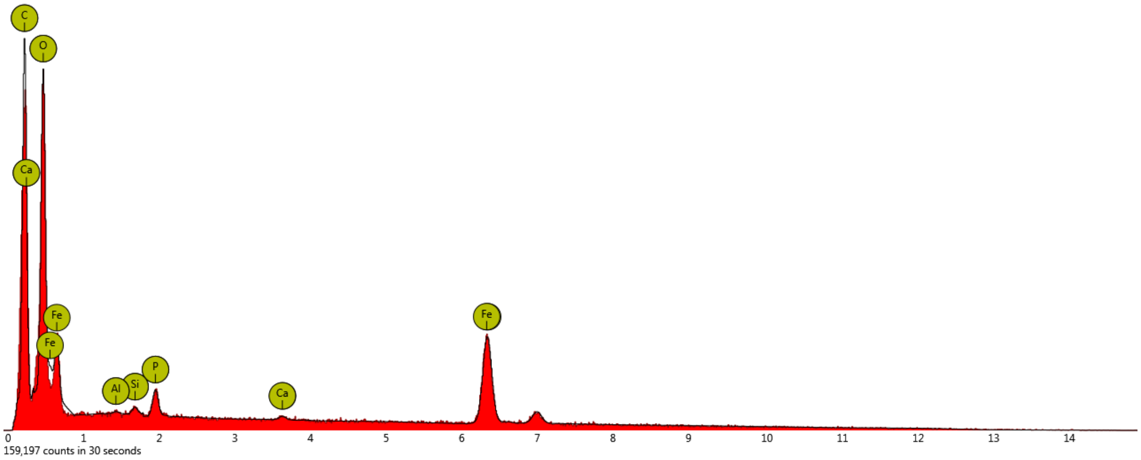

Disabled elements: B

Element Number	Element Symbol	Element Name	Atomic Concentration	Error
6	C	Carbon	19.3	1.6
8	O	Oxygen	66.8	0.0
26	Fe	Iron	11.7	0.0
15	P	Phosphorus	1.5	0.3
14	Si	Silicon	0.4	0.5
20	Ca	Calcium	0.2	0.8
13	Al	Aluminium	0.1	0.6

4. spot

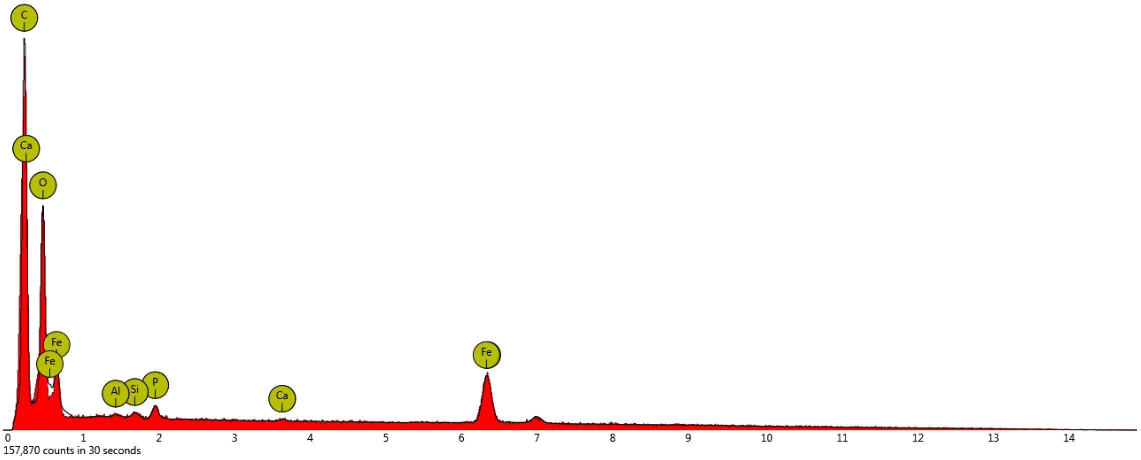

Disabled elements: B

Element Number	Element Symbol	Element Name	Atomic Concentration	Error

6	C	Carbon	26.7	1.5
8	O	Oxygen	62.6	0.0
26	Fe	Iron	9.0	0.0
15	P	Phosphorus	1.0	0.7
14	Si	Silicon	0.3	0.8
20	Ca	Calcium	0.2	0.8
13	Al	Aluminium	0.2	0.4

"Osteocyte-like microstructure" 03 (4 spots marked with cross and number)

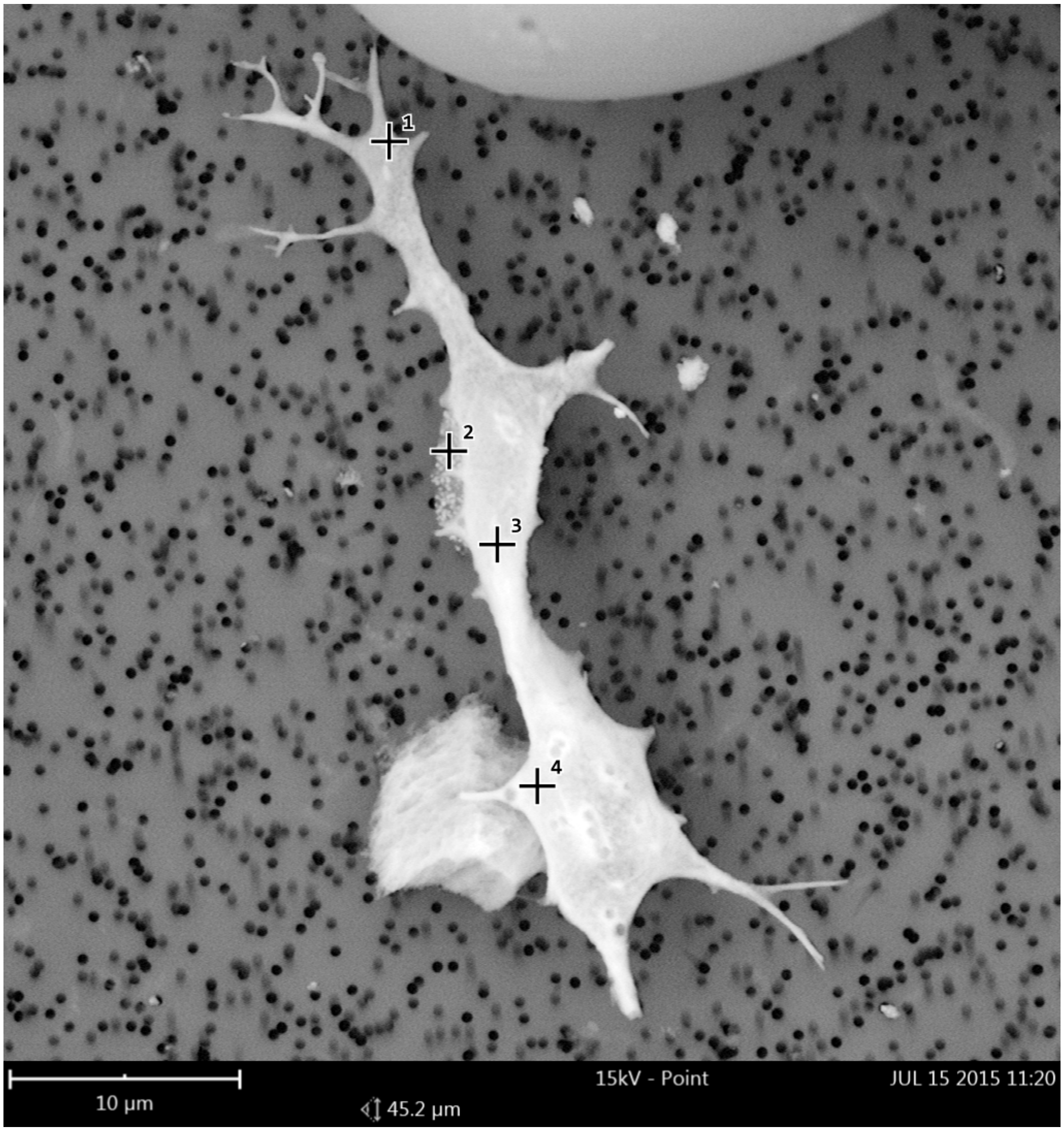

1. spot

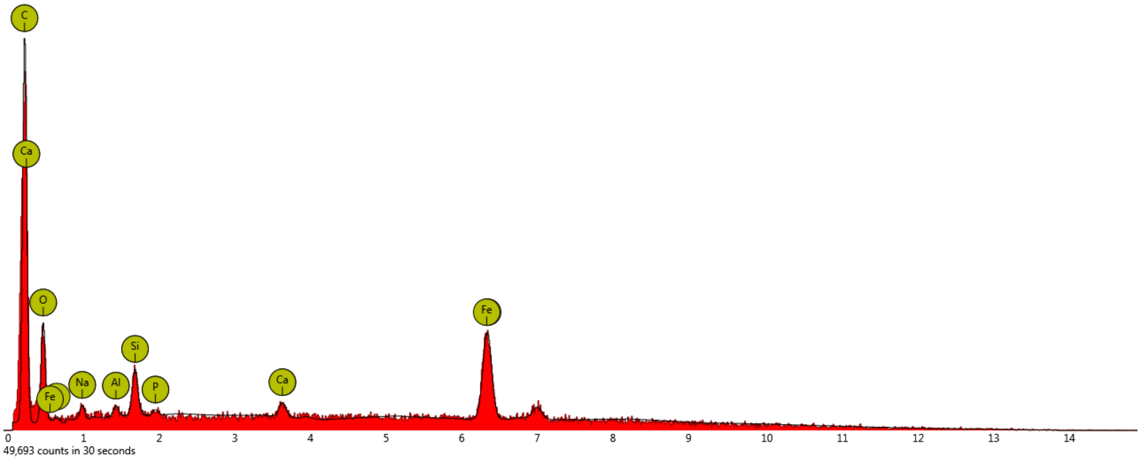

Disabled elements: B

Element Number	Element Symbol	Element Name	Atomic Concentration	Error
6	C	Carbon	34.8	1.9
26	Fe	Iron	15.8	0.2
8	O	Oxygen	39.4	0.4
14	Si	Silicon	3.3	0.1
20	Ca	Calcium	1.1	0.9
11	Na	Sodium	4.1	0.5
13	Al	Aluminium	1.0	0.3
15	P	Phosphorus	0.4	2.4

2. spot

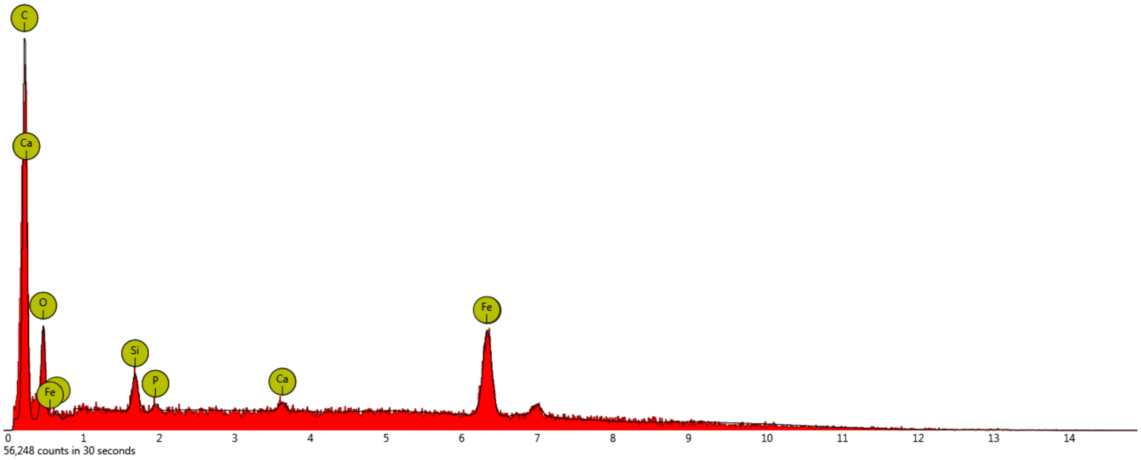

Disabled elements: B

Element Number	Element Symbol	Element Name	Atomic Concentration	Error

6	C	Carbon	36.9	2.6
26	Fe	Iron	17.6	0.1
8	O	Oxygen	41.1	0.2
14	Si	Silicon	3.0	0.4
20	Ca	Calcium	0.8	0.9
15	P	Phosphorus	0.7	0.9

3. spot

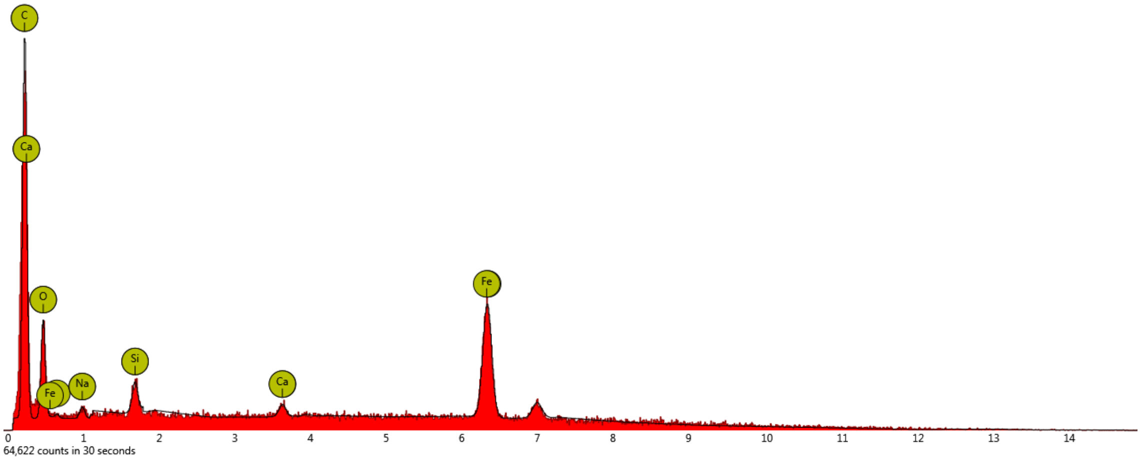

Disabled elements: B

Element Number	Element Symbol	Element Name	Atomic Concentration	Error
6	C	Carbon	34.2	2.8
26	Fe	Iron	21.2	0.1
8	O	Oxygen	37.6	0.1
14	Si	Silicon	2.3	0.6
20	Ca	Calcium	1.0	0.5
11	Na	Sodium	3.7	1.2

4. spot

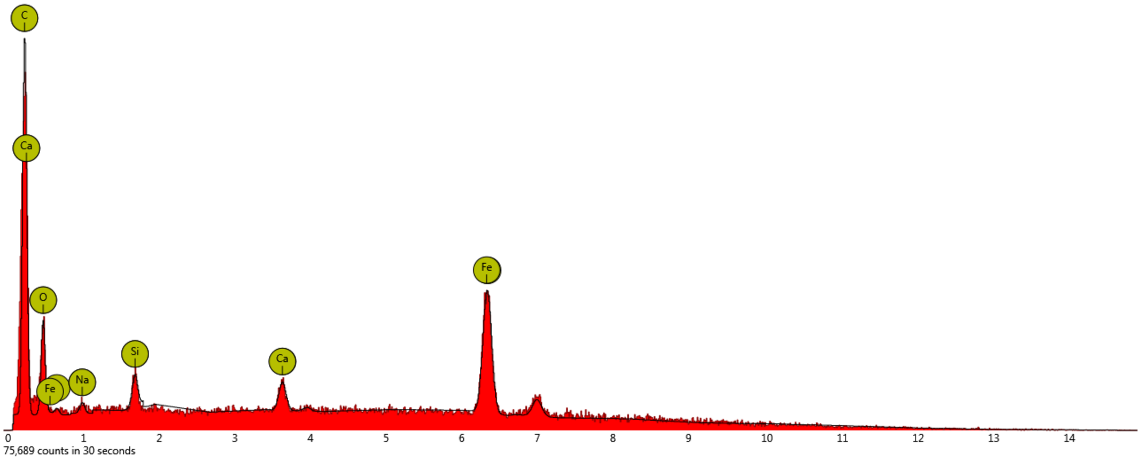

Disabled elements: Ag, B

Element Number	Element Symbol	Element Name	Atomic Concentration	Error
6	C	Carbon	33.0	2.2
26	Fe	Iron	22.2	0.1
8	O	Oxygen	36.7	0.1
20	Ca	Calcium	2.2	0.4
14	Si	Silicon	2.4	0.7
11	Na	Sodium	3.5	0.8

“Osteocyte-like microstructure” 04 (4 spots marked with cross and number)

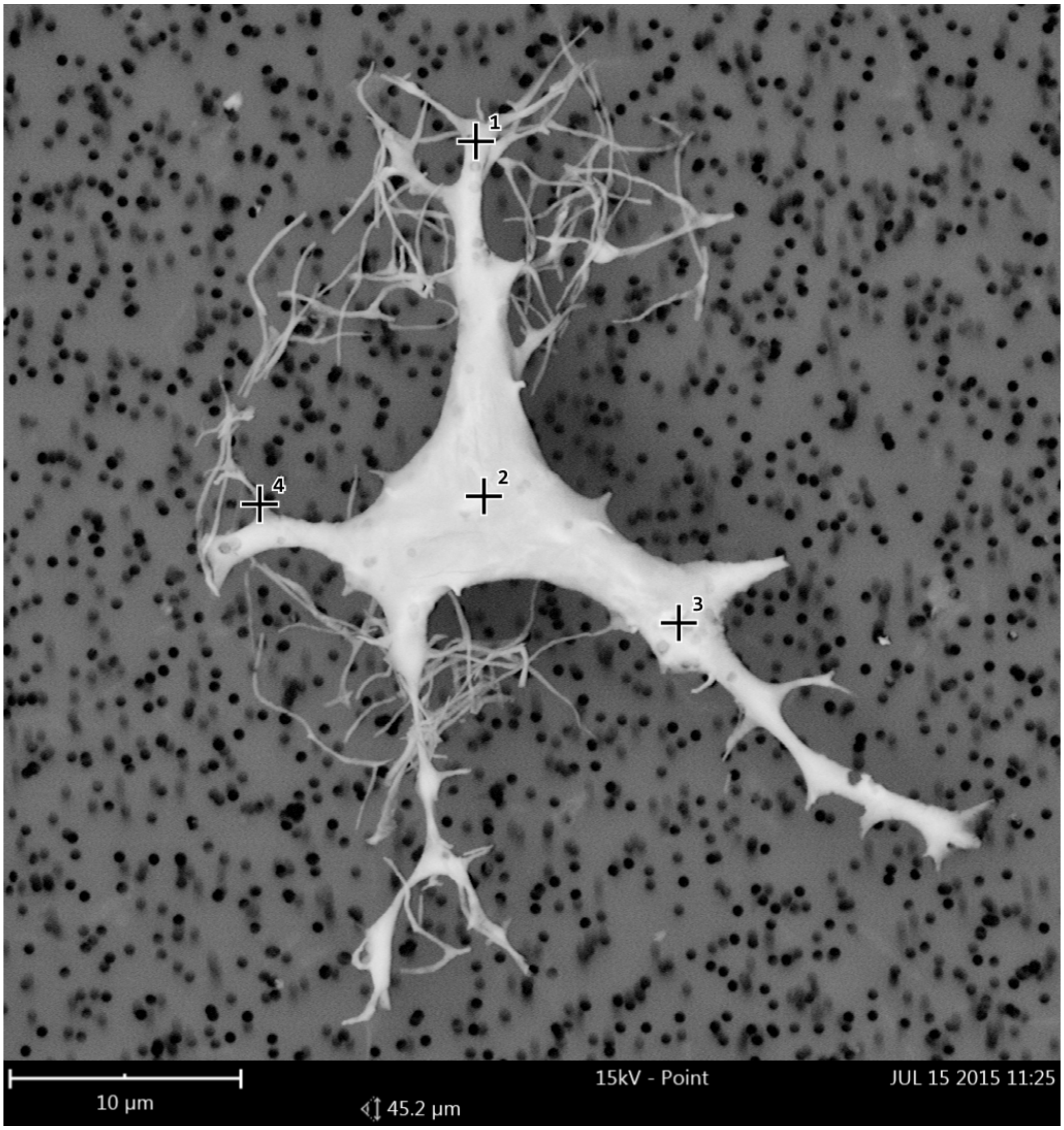

1. spot

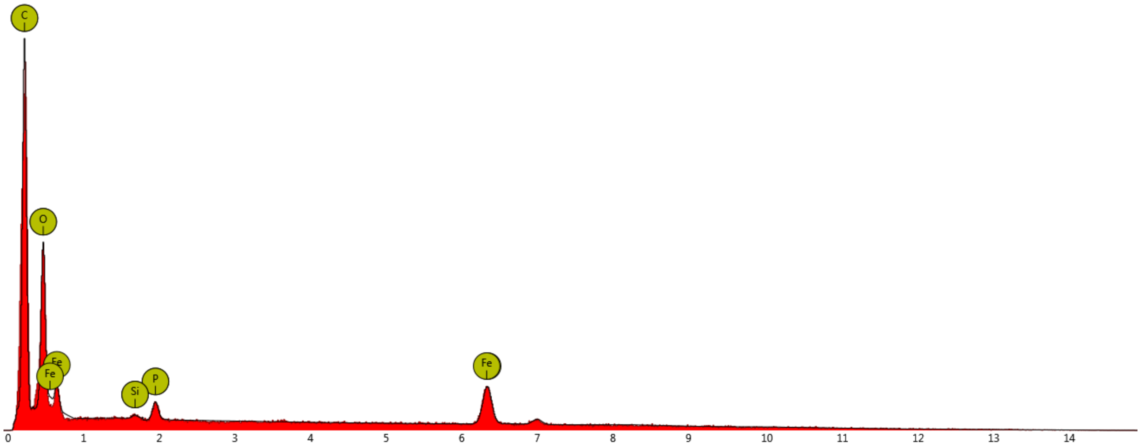

Disabled elements: B

Element Number	Element Symbol	Element Name	Atomic Concentration	Error
6	C	Carbon	29.2	1.1
8	O	Oxygen	61.2	0.0
26	Fe	Iron	7.9	0.1
15	P	Phosphorus	1.5	0.2
14	Si	Silicon	0.3	1.0

2. spot

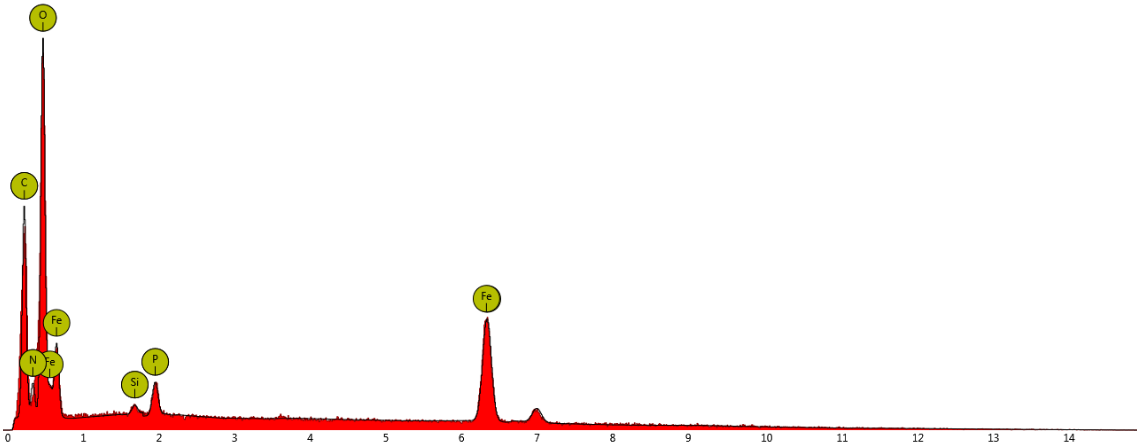

Disabled elements: B

Element Number	Element Symbol	Element Name	Atomic Concentration	Error
8	O	Oxygen	69.4	0.1
26	Fe	Iron	12.3	0.1

6	C	Carbon	10.6	1.6
15	P	Phosphorus	1.6	0.4
7	N	Nitrogen	5.7	1.6
14	Si	Silicon	0.4	1.0

3. spot

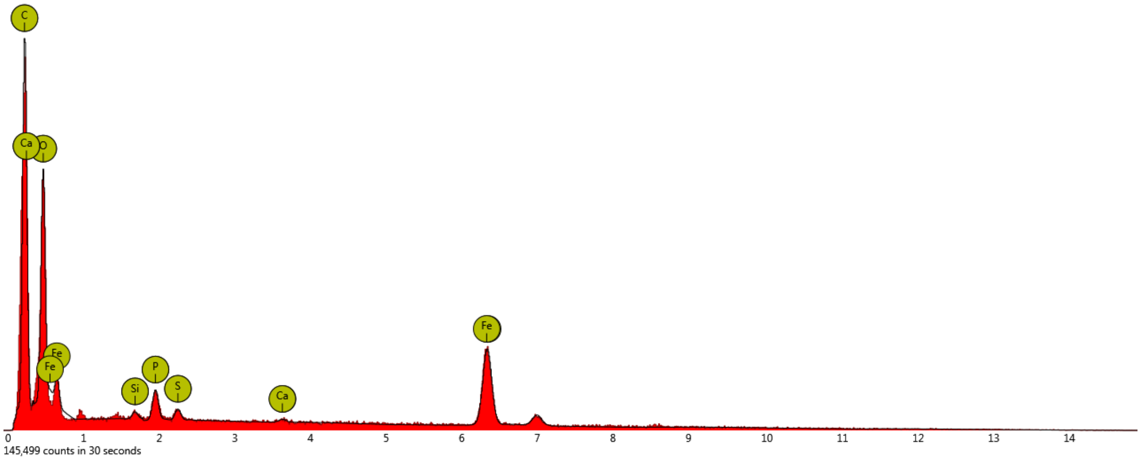

Disabled elements: B

Element Number	Element Symbol	Element Name	Atomic Concentration	Error
6	C	Carbon	24.7	1.5
8	O	Oxygen	60.9	0.0
26	Fe	Iron	11.4	0.1
15	P	Phosphorus	1.8	0.2
16	S	Sulfur	0.6	0.6
14	Si	Silicon	0.4	0.8
20	Ca	Calcium	0.2	1.0

4. spot

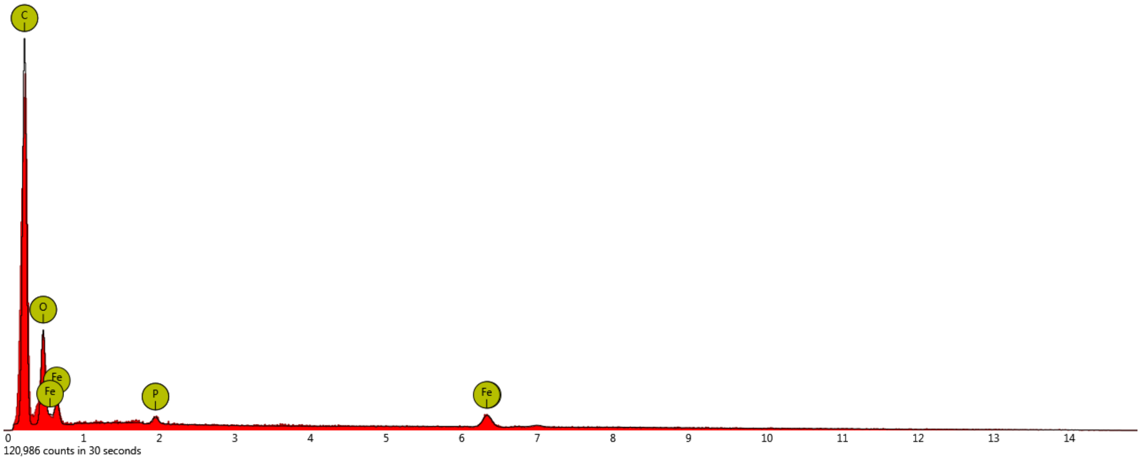

Disabled elements: B

Element Number	Element Symbol	Element Name	Atomic Concentration	Error

6	C	Carbon	36.8	1.5
8	O	Oxygen	58.7	0.1
26	Fe	Iron	3.7	0.2
15	P	Phosphorus	0.8	0.6

Bone matrix fragment (3 spots marked with cross and number)

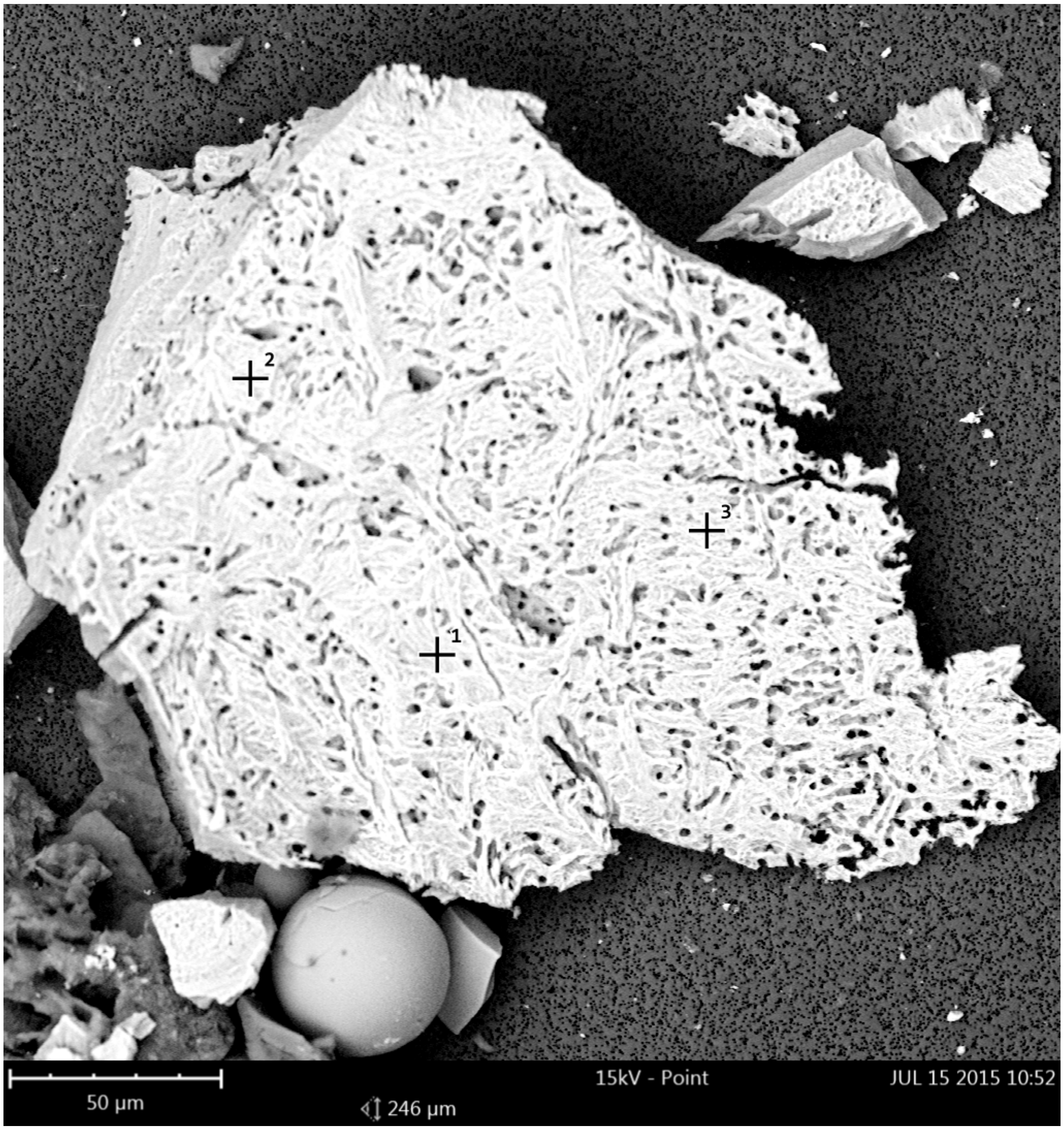

1. spot

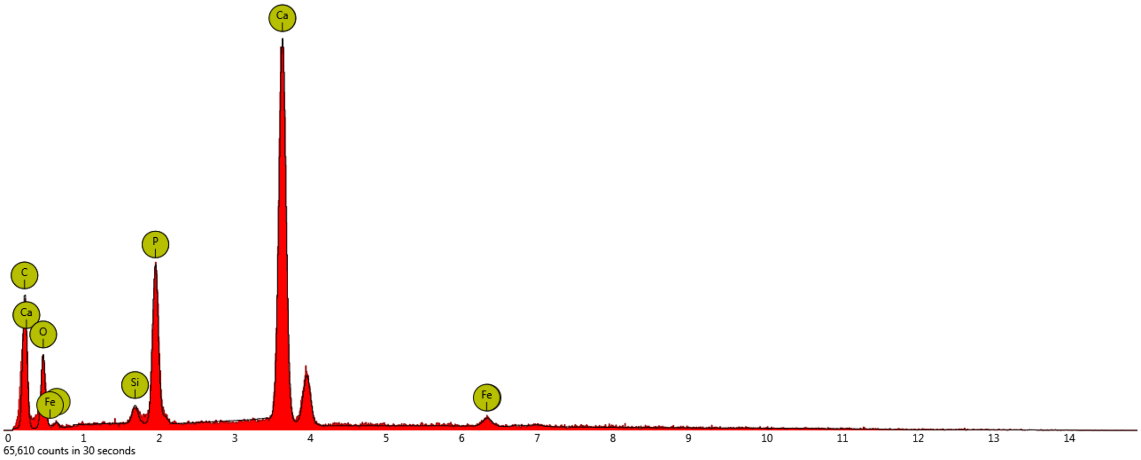

Disabled elements: B, Sr, Ti

Element Number	Element Symbol	Element Name	Atomic Concentration	Error
20	Ca	Calcium	25.6	0.0
15	P	Phosphorus	8.7	0.1
6	C	Carbon	10.4	1.1
8	O	Oxygen	53.2	0.1
14	Si	Silicon	0.9	7.7
26	Fe	Iron	1.3	0.4

2. spot

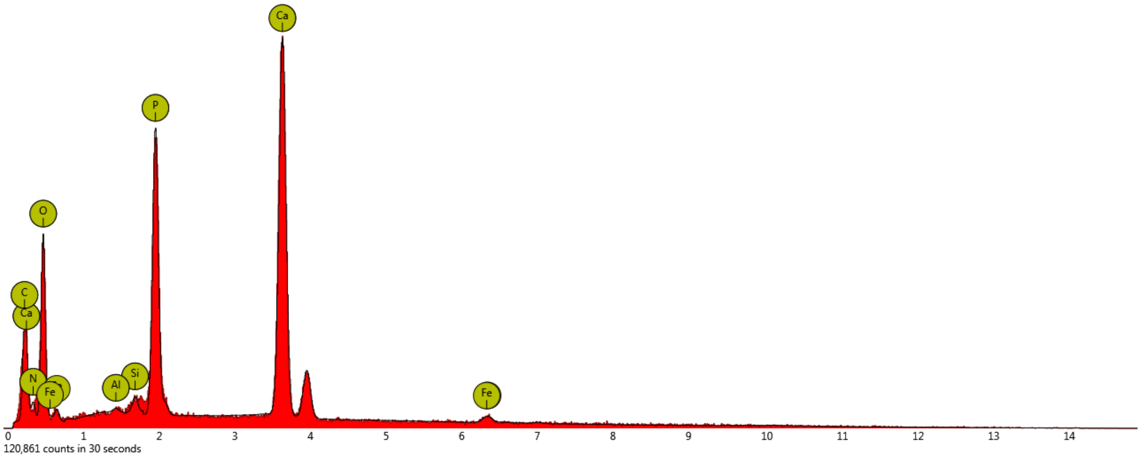

Element Number	Element Symbol	Element Name	Atomic Concentration	Error
20	Ca	Calcium	14.6	0.1
15	P	Phosphorus	8.9	0.1
8	O	Oxygen	63.3	0.1

6	C	Carbon	5.1	2.2
7	N	Nitrogen	7.0	2.7
14	Si	Silicon	0.4	11.9
26	Fe	Iron	0.6	0.7
13	Al	Aluminium	0.1	0.7

3. spot

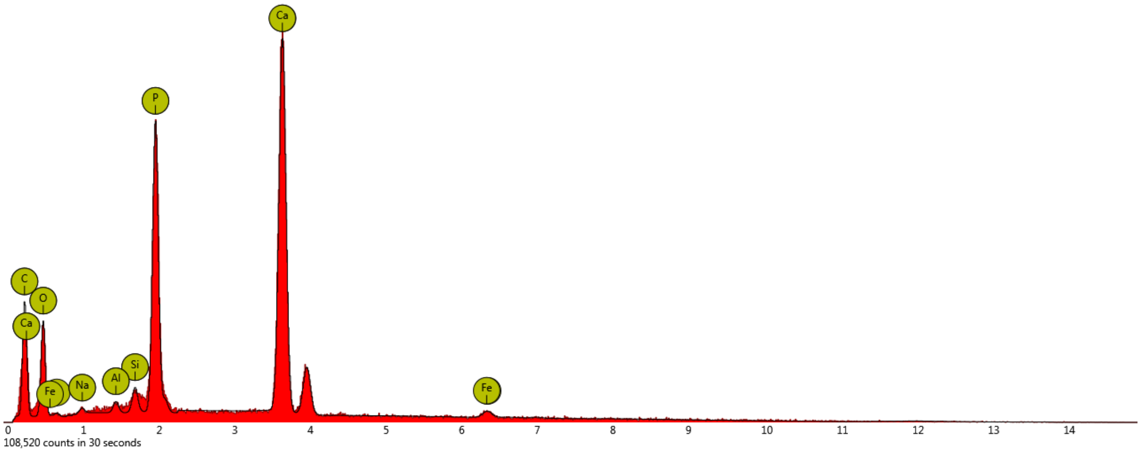

Disabled elements: Br, Rb, Sr

Element Number	Element Symbol	Element Name	Atomic Concentration	Error
20	Ca	Calcium	21.2	0.0
15	P	Phosphorus	13.2	0.1
6	C	Carbon	8.2	1.8
8	O	Oxygen	54.1	0.0
14	Si	Silicon	1.0	14.4
13	Al	Aluminium	0.6	2.6
26	Fe	Iron	0.8	0.6
11	Na	Sodium	0.8	0.3
