# Supplementary material for: Microscopical and elemental FESEM and Phenom ProX-SEM-EDS analysis of osteocyte- and blood vessel-like microstructures obtained from fossil vertebrates of the Eocene Messel Pit, Germany
Source: PeerJ. 2016 Jan 21;4:e1618. doi: 10.7717/peerj.1618 (PMC4727973; doi:10.7717/peerj.1618)
Supplement: Supplemental Information 2 [file peerj-04-1618-s002.pdf]

Supplementary information 2

Elemental analyses of OBvF-like microstructures of *Neochelys franzeni* SMF ME 1091 conducted using a Phenom ProX desktop scanning electron microscope (LOT-QuantumDesign) equipped with a thermionic CeB6 source and a high sensitivity multi-mode backscatter electron (BSE) detector, 0.15 kV EHT (primary-beam energy), also at the Geosciences Department, Goethe Universität, Frankfurt, Germany.

“Osteocyte-like microstructure” 01 (5 spots marked with cross and number)

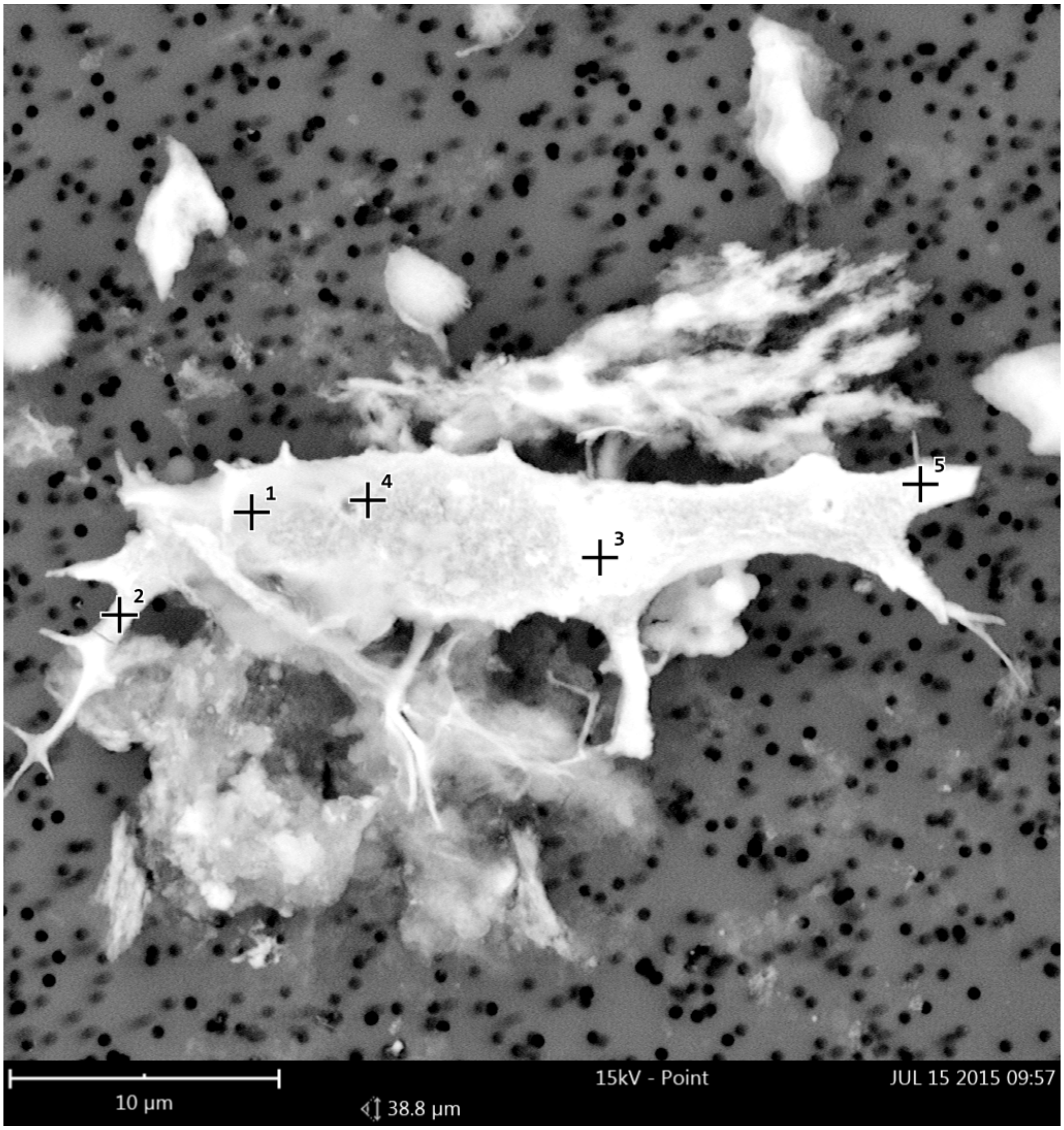

1. spot

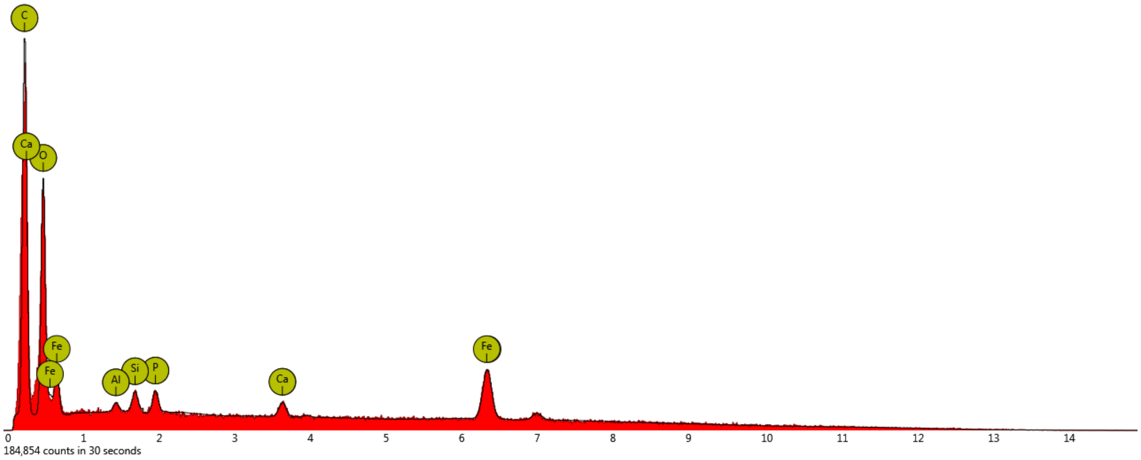

Disabled elements: B

**Element Number**	**Element Symbol**	**Element Name**	**Atomic Concentration**	**Error**
6	C	Carbon	23.7	2.1
8	O	Oxygen	64.6	0.0
26	Fe	Iron	7.7	0.1
15	P	Phosphorus	1.3	0.2
14	Si	Silicon	1.1	0.2
20	Ca	Calcium	0.9	0.3
13	Al	Aluminium	0.6	0.1

2. spot

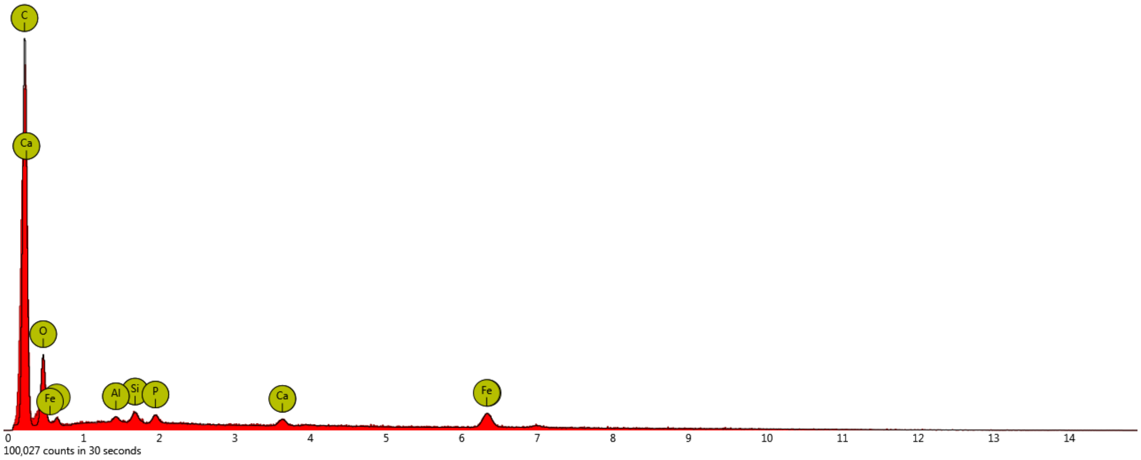

Disabled elements: B

**Element Number**	**Element Symbol**	**Element Name**	**Atomic Concentration**	**Error**
6	C	Carbon	41.5	1.6
8	O	Oxygen	50.8	0.0
26	Fe	Iron	4.3	0.1
14	Si	Silicon	1.0	0.4

15	P	Phosphorus	0.9	0.3
20	Ca	Calcium	0.8	0.4
13	Al	Aluminium	0.7	0.1

3. spot

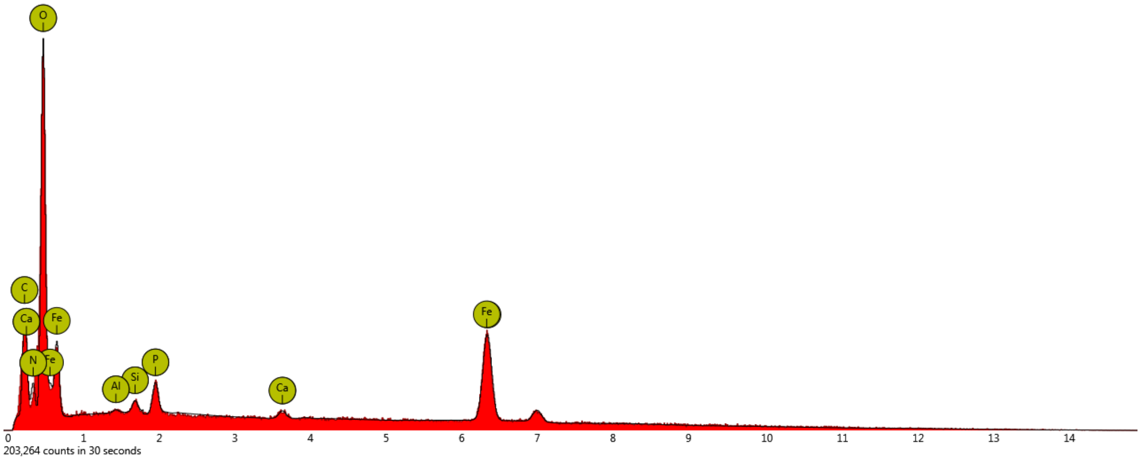

Disabled elements: Te

Element Number	Element Symbol	Element Name	Atomic Concentration	Error
8	O	Oxygen	73.8	0.0
26	Fe	Iron	11.4	0.1
6	C	Carbon	6.2	1.2
15	P	Phosphorus	1.6	0.1
7	N	Nitrogen	5.8	1.4
14	Si	Silicon	0.6	0.4
20	Ca	Calcium	0.4	1.0
13	Al	Aluminium	0.2	0.4

4. spot

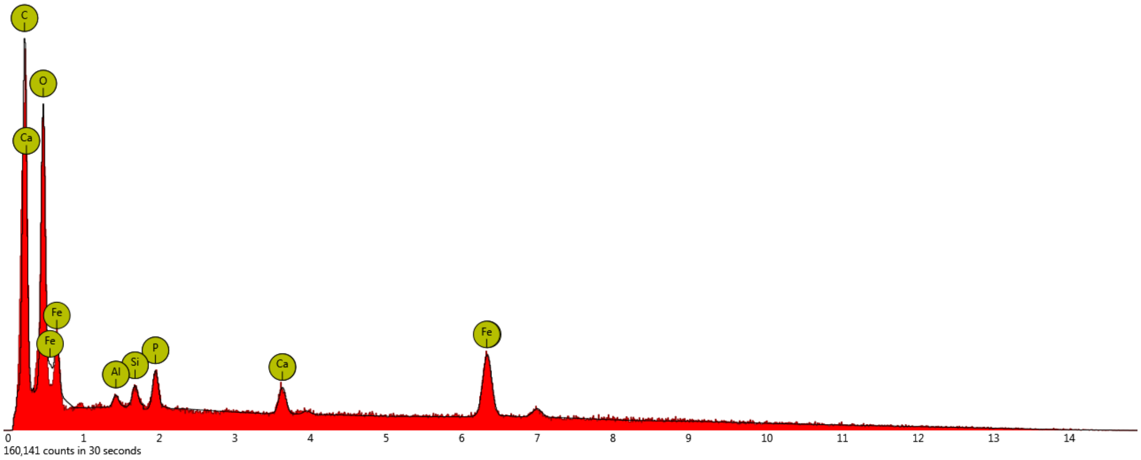

Element Number	Element Symbol	Element Name	Atomic Concentration	Error
6	C	Carbon	20.1	1.8

8	O	Oxygen	66.5	0.0
26	Fe	Iron	8.2	0.1
15	P	Phosphorus	2.0	0.2
20	Ca	Calcium	1.5	0.4
14	Si	Silicon	1.0	0.4
13	Al	Aluminium	0.8	0.3

5. spot

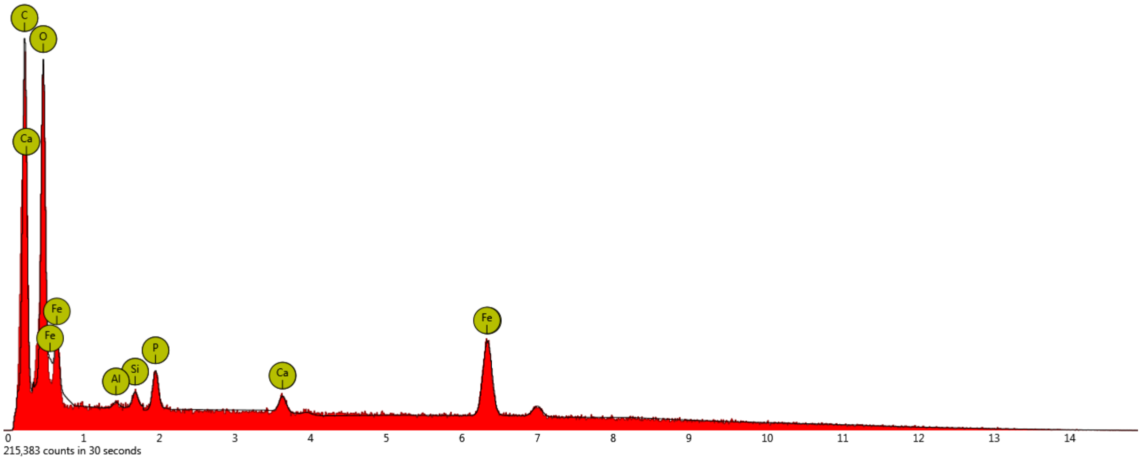

Disabled elements: Te

Element Number	Element Symbol	Element Name	Atomic Concentration	Error
6	C	Carbon	19.3	1.0
8	O	Oxygen	67.3	0.0
26	Fe	Iron	9.6	0.1
15	P	Phosphorus	2.0	0.1
20	Ca	Calcium	0.9	0.5
14	Si	Silicon	0.7	0.3
13	Al	Aluminium	0.4	0.6

“Osteocyte-like microstructure” 02 (3 spots marked with cross and number)

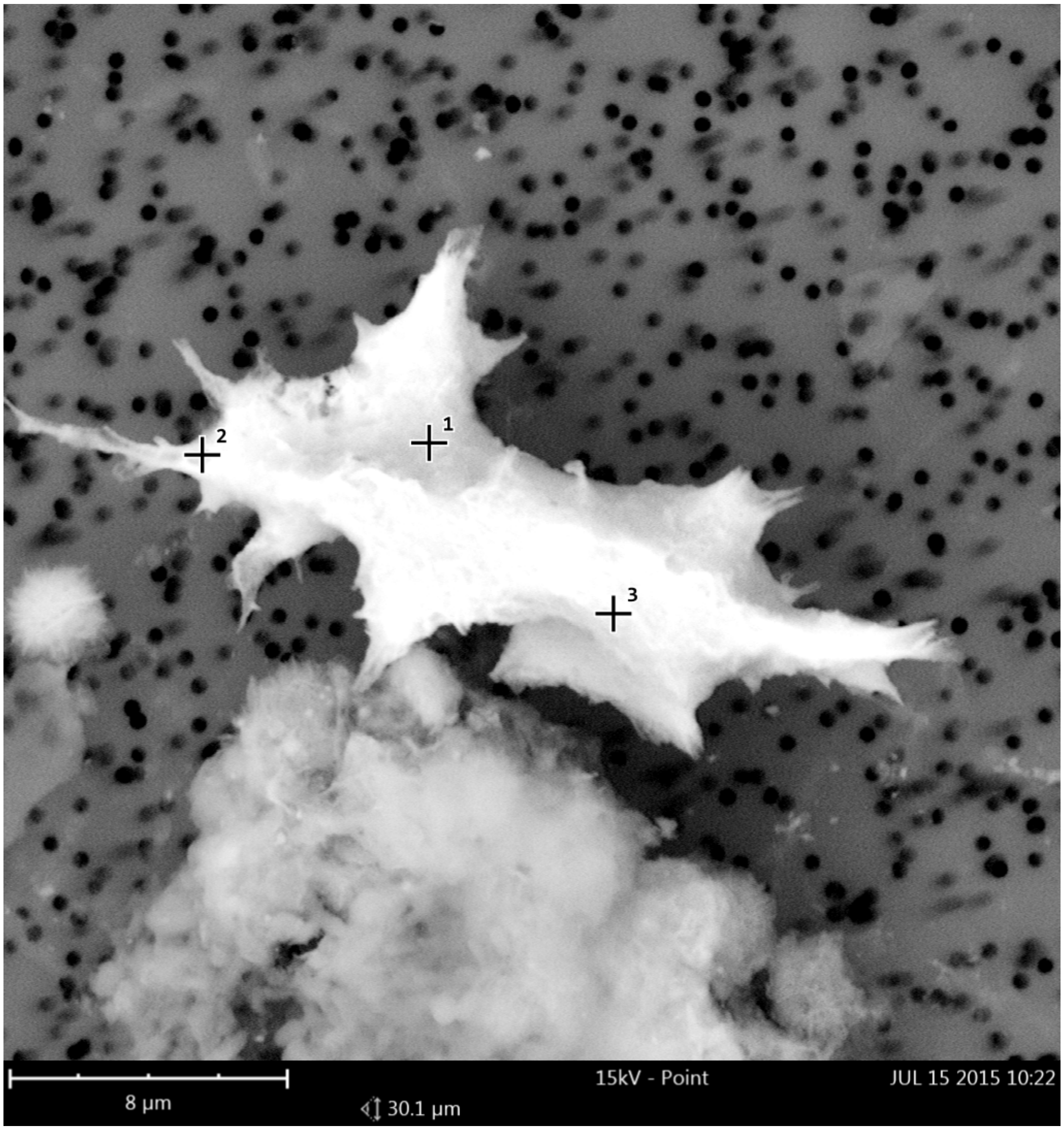

1. spot

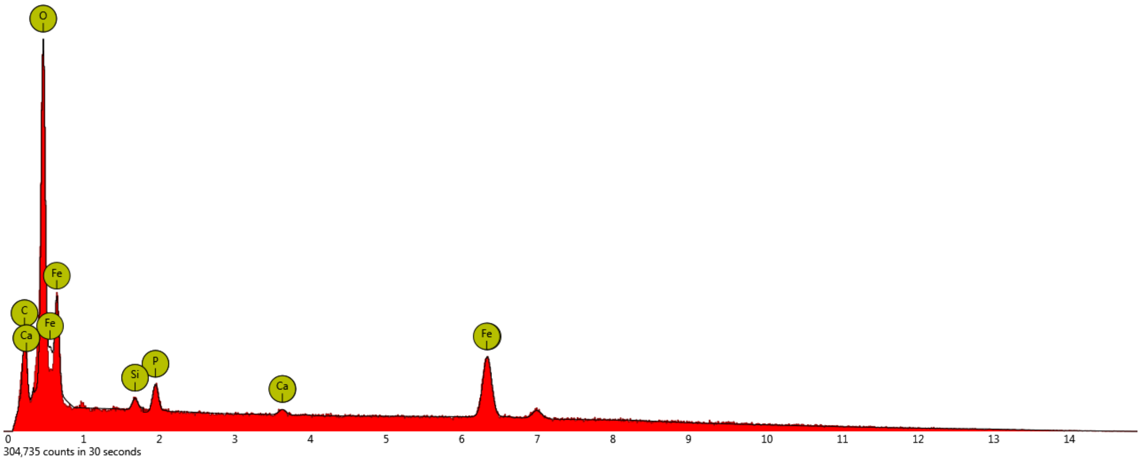

Element Number	Element Symbol	Element Name	Atomic Concentration	Error
8	O	Oxygen	79.7	0.0
26	Fe	Iron	11.2	0.1
6	C	Carbon	5.7	1.1
15	P	Phosphorus	2.1	0.1
14	Si	Silicon	0.8	0.7
20	Ca	Calcium	0.4	0.6

2. spot

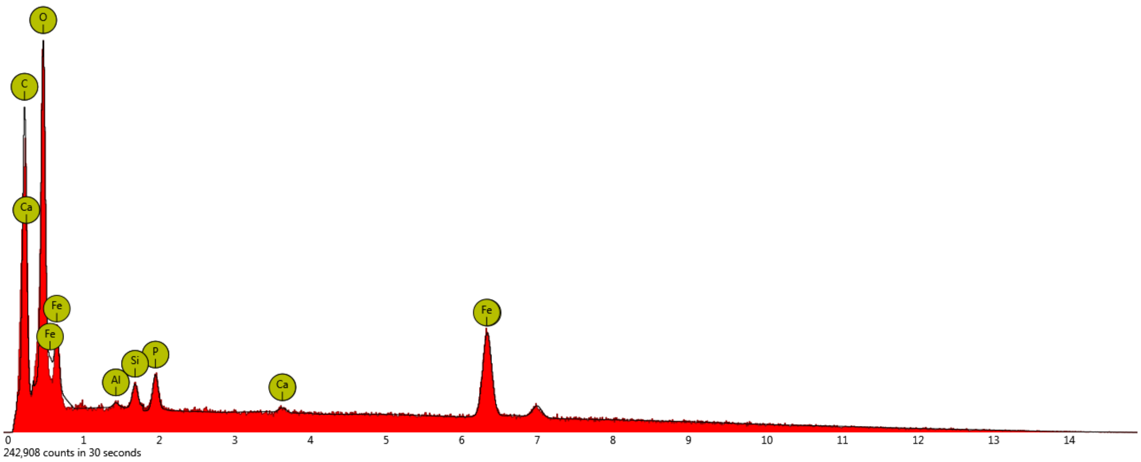

Element Number	Element Symbol	Element Name	Atomic Concentration	Error
8	O	Oxygen	68.8	0.0
6	C	Carbon	16.2	1.3
26	Fe	Iron	11.2	0.0
15	P	Phosphorus	2.0	0.1
14	Si	Silicon	1.2	0.3
20	Ca	Calcium	0.3	0.8

13	Al	Aluminium	0.3	0.4

3. spot

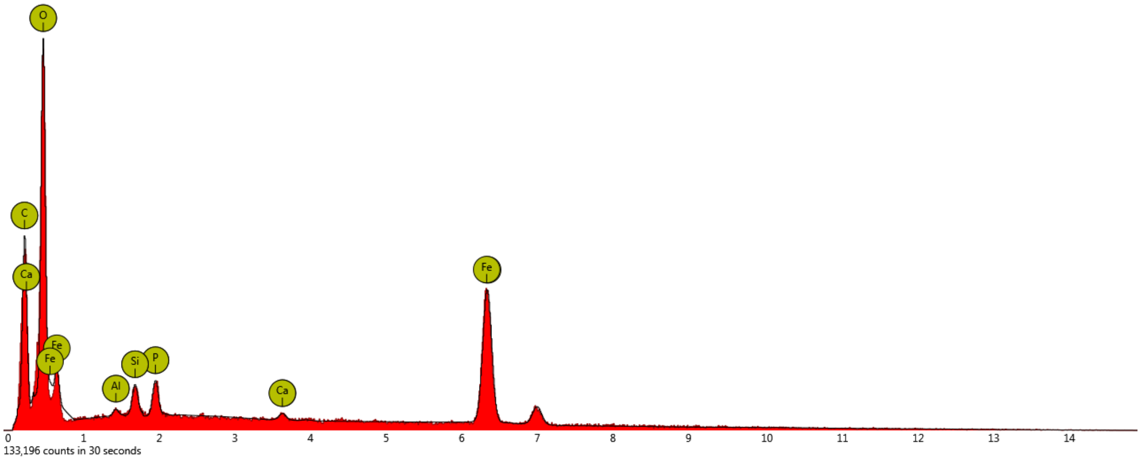

Disabled elements: B

Element Number	Element Symbol	Element Name	Atomic Concentration	Error
8	O	Oxygen	67.2	0.0
26	Fe	Iron	17.3	0.1
6	C	Carbon	11.0	1.3
15	P	Phosphorus	2.0	0.3
14	Si	Silicon	1.6	0.3
20	Ca	Calcium	0.4	0.6
13	Al	Aluminium	0.5	0.4

“blood vessel-like microstructure” 01 (3 spots marked with cross and number)

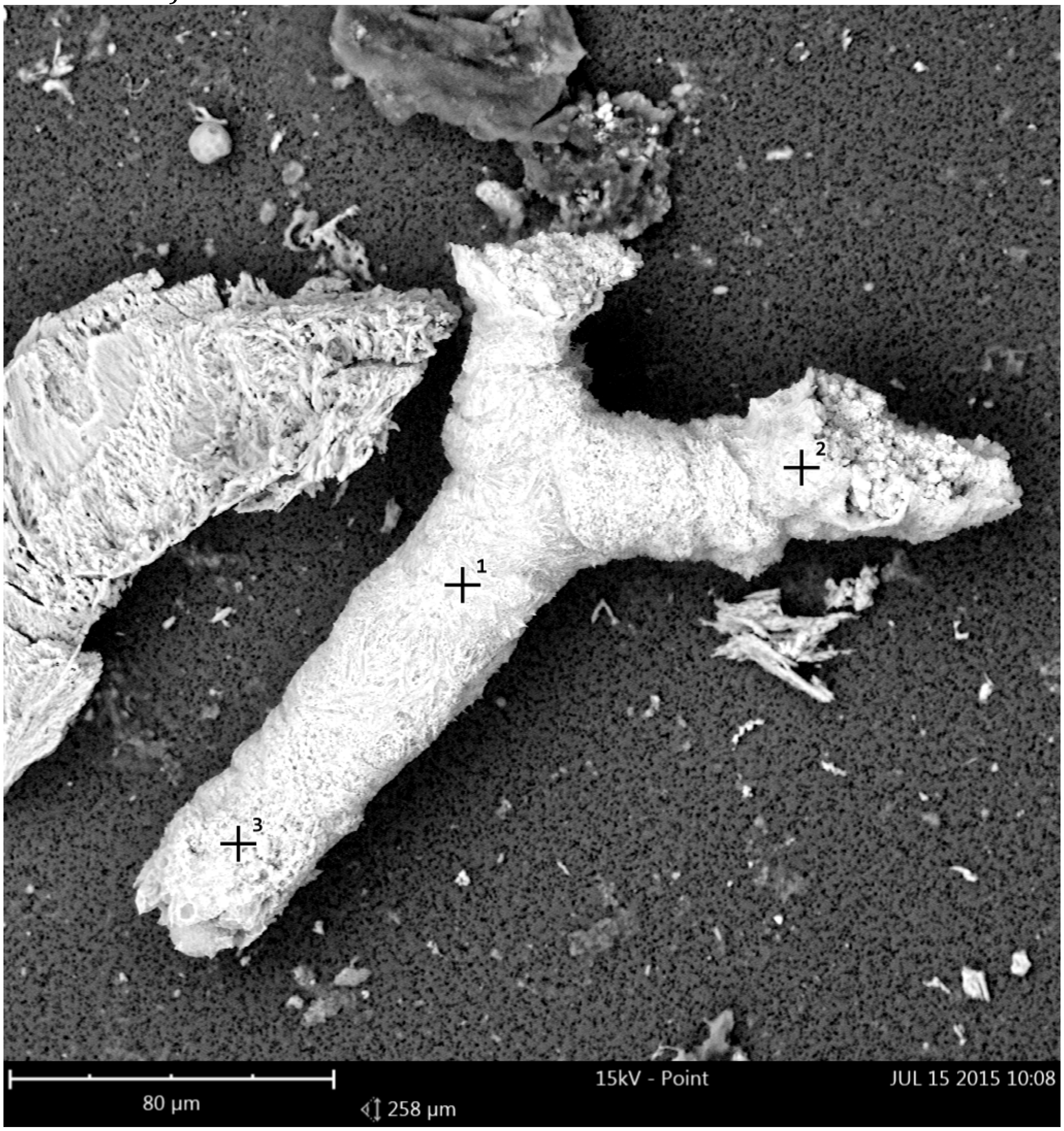

1. spot

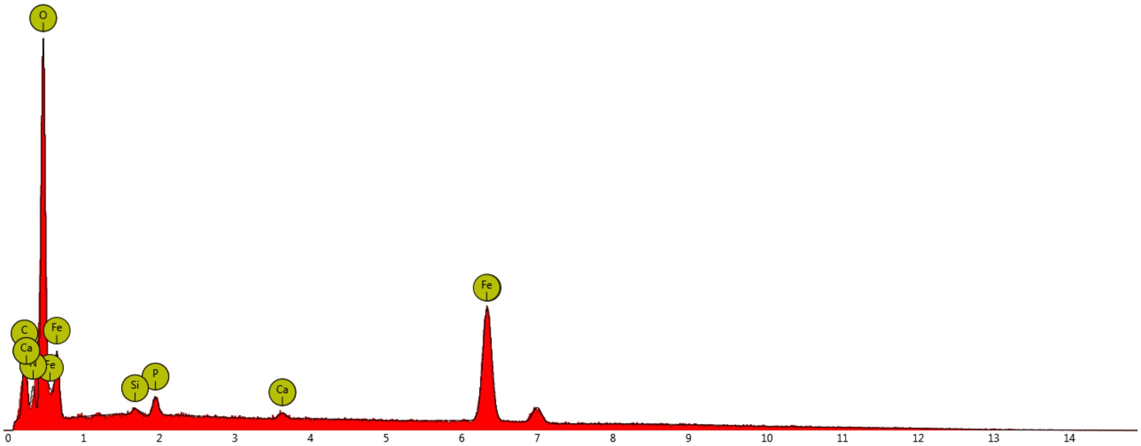

Element Number	Element Symbol	Element Name	Atomic Concentration	Error
8	O	Oxygen	73.1	0.0
26	Fe	Iron	15.6	0.0
6	C	Carbon	4.1	1.1
7	N	Nitrogen	5.5	1.4
15	P	Phosphorus	1.1	0.3
20	Ca	Calcium	0.4	1.0
14	Si	Silicon	0.3	0.8

2. spot

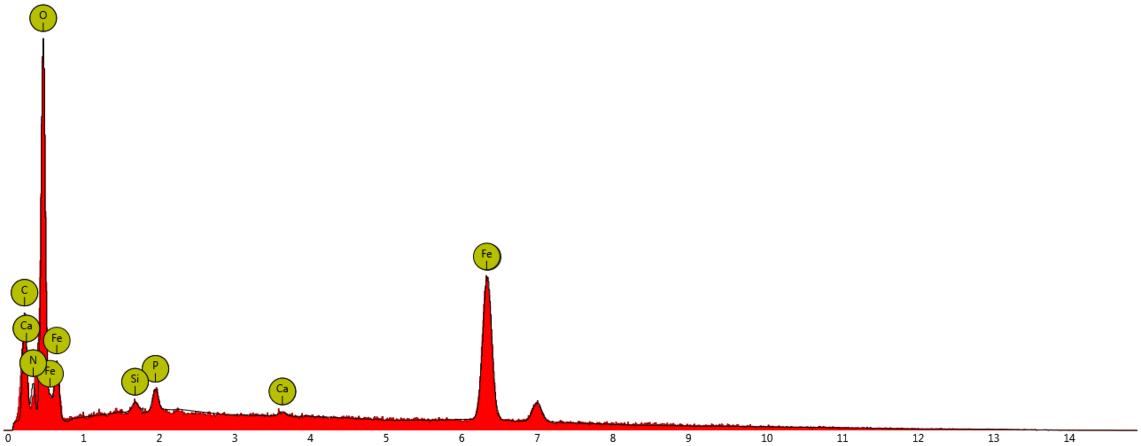

Disabled elements: B

Element Number	Element Symbol	Element Name	Atomic Concentration	Error
8	O	Oxygen	68.7	0.0
26	Fe	Iron	17.5	0.1
6	C	Carbon	6.0	2.2

7	N	Nitrogen	6.0	1.5
15	P	Phosphorus	1.2	0.2
14	Si	Silicon	0.4	0.7
20	Ca	Calcium	0.2	1.2

3. spot

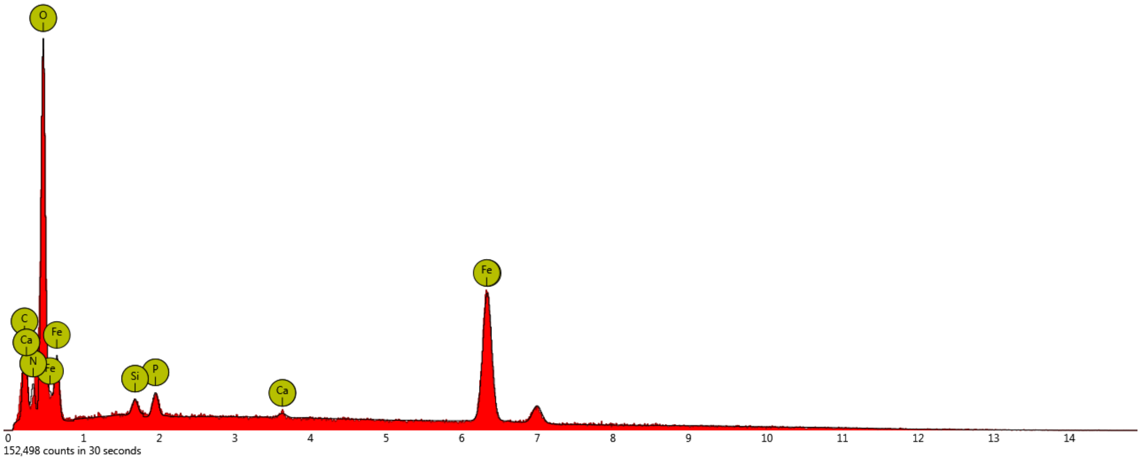

Element Number	Element Symbol	Element Name	Atomic Concentration	Error
8	O	Oxygen	70.6	0.0
26	Fe	Iron	16.3	0.1
6	C	Carbon	4.7	1.1
7	N	Nitrogen	6.0	1.5
15	P	Phosphorus	1.3	0.8
14	Si	Silicon	0.8	0.9
20	Ca	Calcium	0.2	0.7

“collagen fibril-like microstructure” 01 (3 spots marked with cross and number)

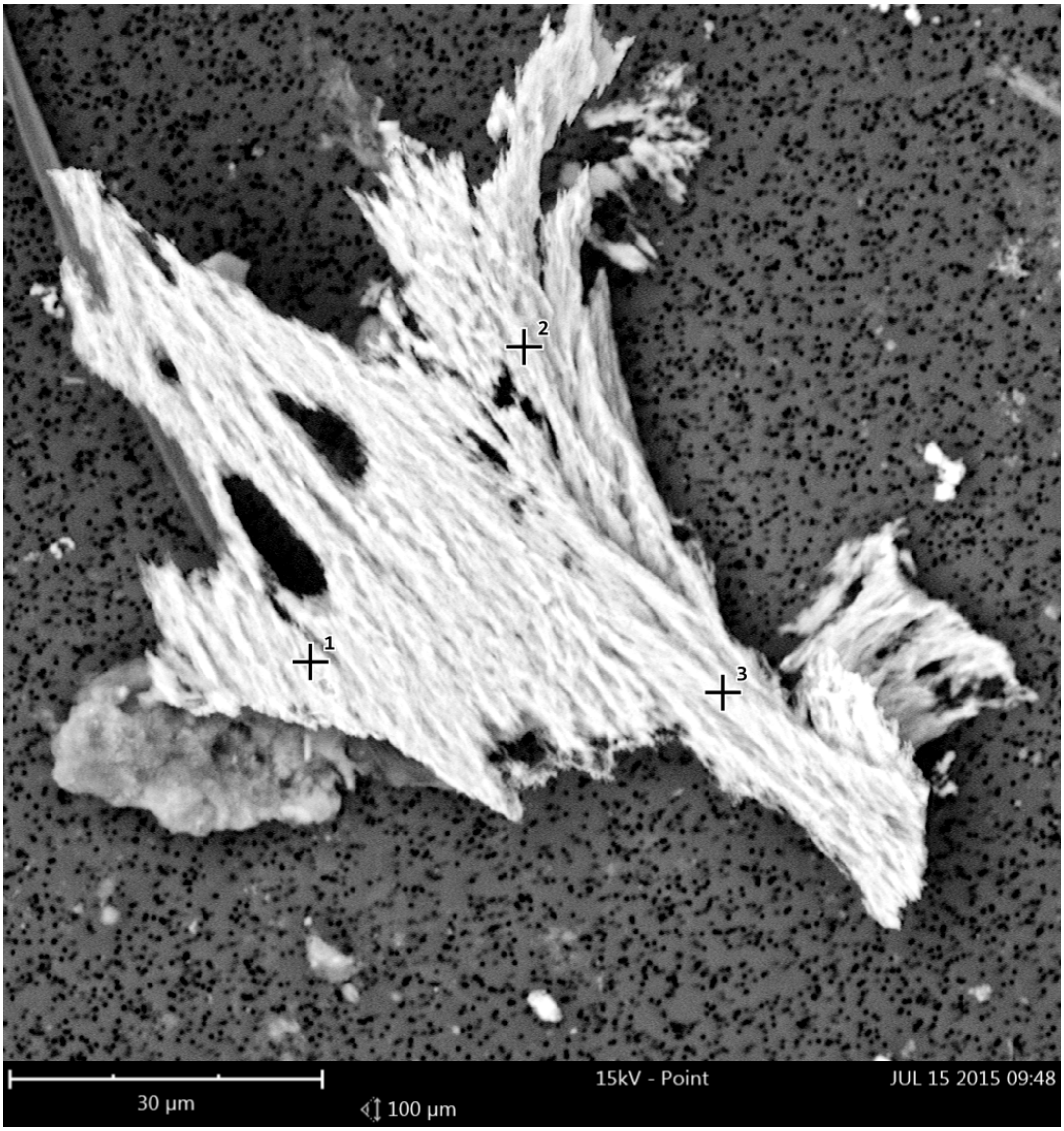

1. spot

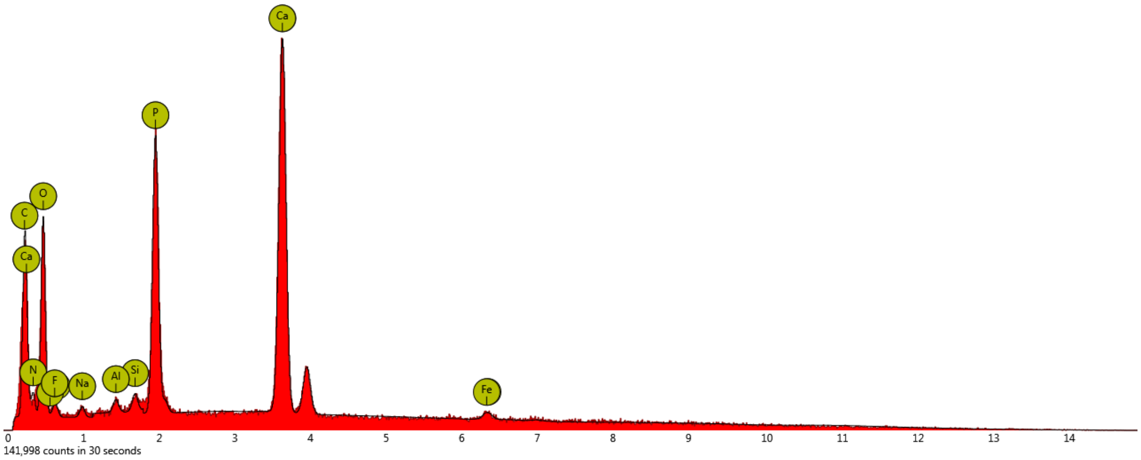

Element Number	Element Symbol	Element Name	Atomic Concentration	Error
20	Ca	Calcium	12.8	0.0
15	P	Phosphorus	7.7	0.1
8	O	Oxygen	58.0	0.0
6	C	Carbon	7.5	1.6
7	N	Nitrogen	7.6	3.6
14	Si	Silicon	0.4	8.0
13	Al	Aluminium	0.4	0.3
26	Fe	Iron	0.6	0.6
9	F	Fluorine	3.9	0.3
11	Na	Sodium	1.1	0.9

2. spot

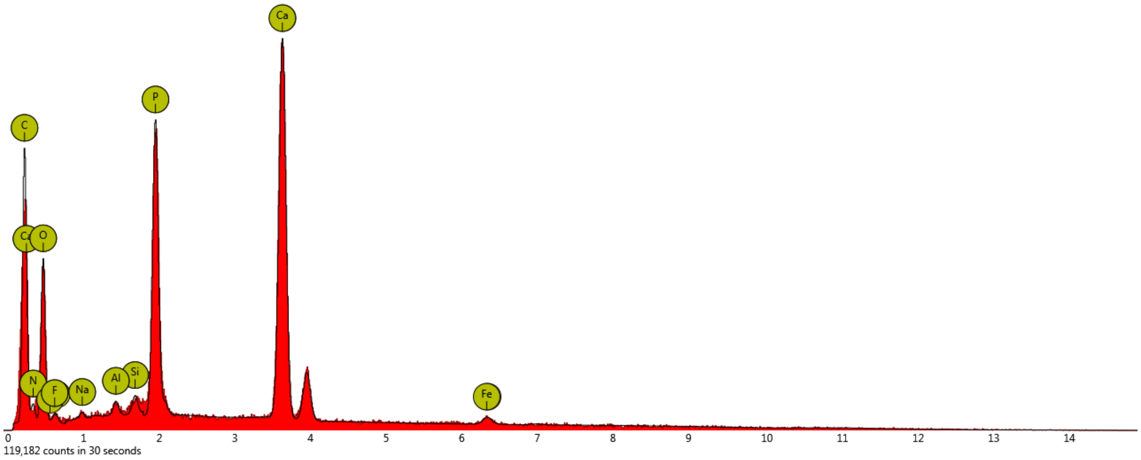

Disabled elements: B

Element Number	Element Symbol	Element Name	Atomic Concentration	Error
20	Ca	Calcium	13.9	0.0
15	P	Phosphorus	8.7	0.1
6	C	Carbon	11.1	2.6
8	O	Oxygen	54.1	0.1
14	Si	Silicon	0.5	15.3
7	N	Nitrogen	6.8	3.8
13	Al	Aluminium	0.5	0.5
26	Fe	Iron	0.6	0.5
9	F	Fluorine	3.1	1.7
11	Na	Sodium	0.6	0.5

3. spot

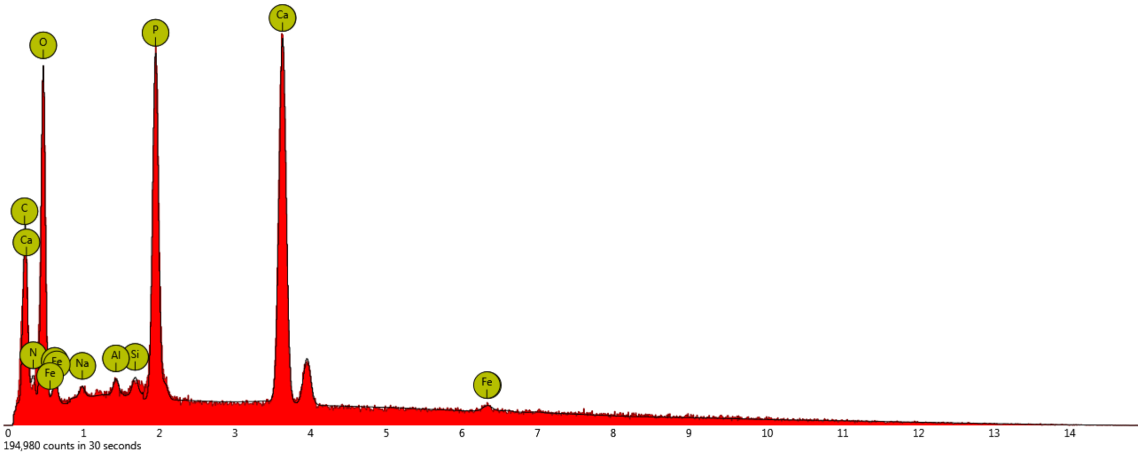

Element Number	Element Symbol	Element Name	Atomic Concentration	Error
20	Ca	Calcium	9.7	0.1
15	P	Phosphorus	7.4	0.1
8	O	Oxygen	64.5	0.0
6	C	Carbon	5.6	1.3
7	N	Nitrogen	5.8	2.7
14	Si	Silicon	0.3	8.4
9	F	Fluorine	5.2	0.1
13	Al	Aluminium	0.4	0.2
11	Na	Sodium	0.8	0.8
26	Fe	Iron	0.3	0.7

“collagen fibril-like microstructure” 02 (3 spots marked with cross and number)

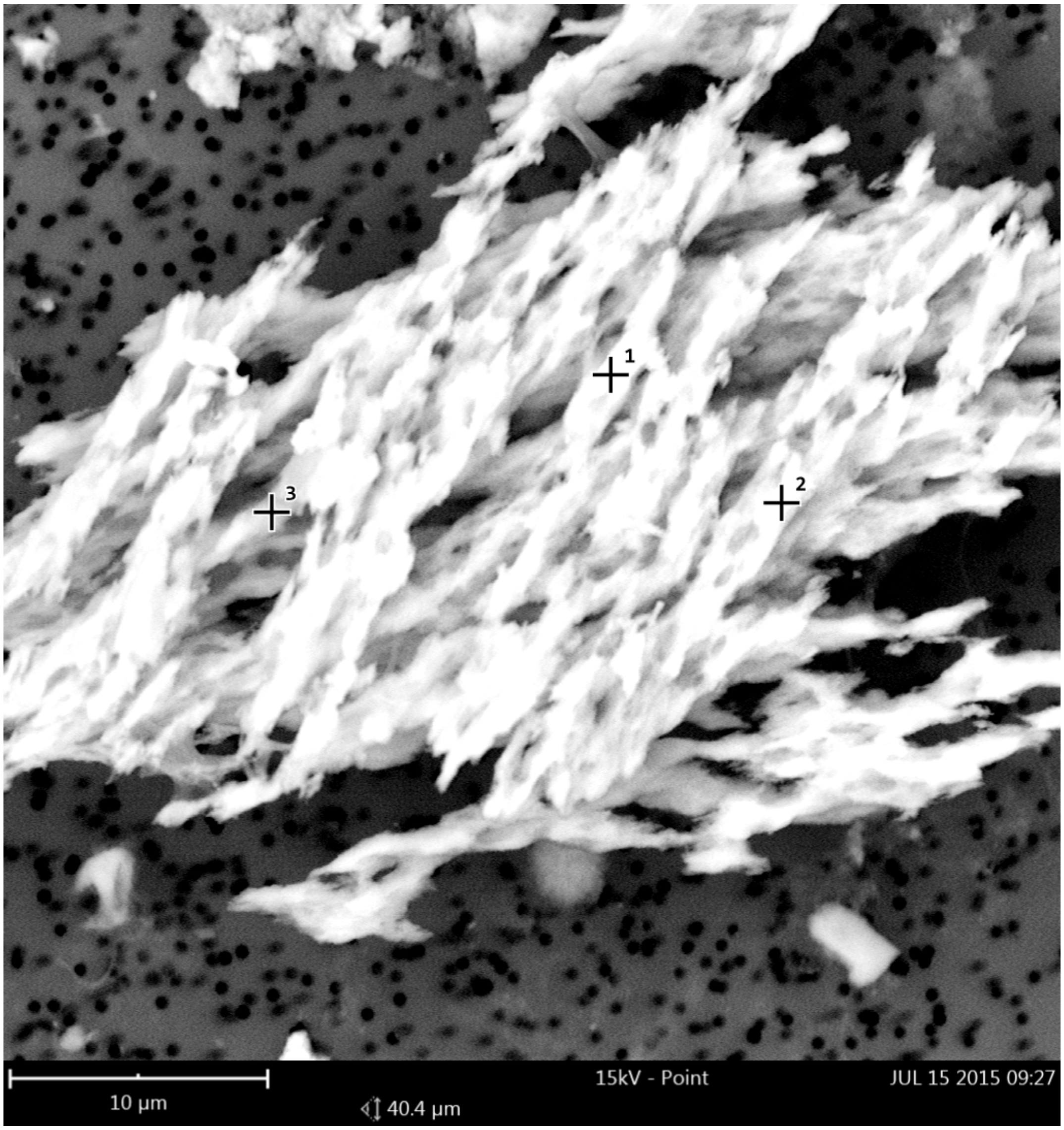

1. spot

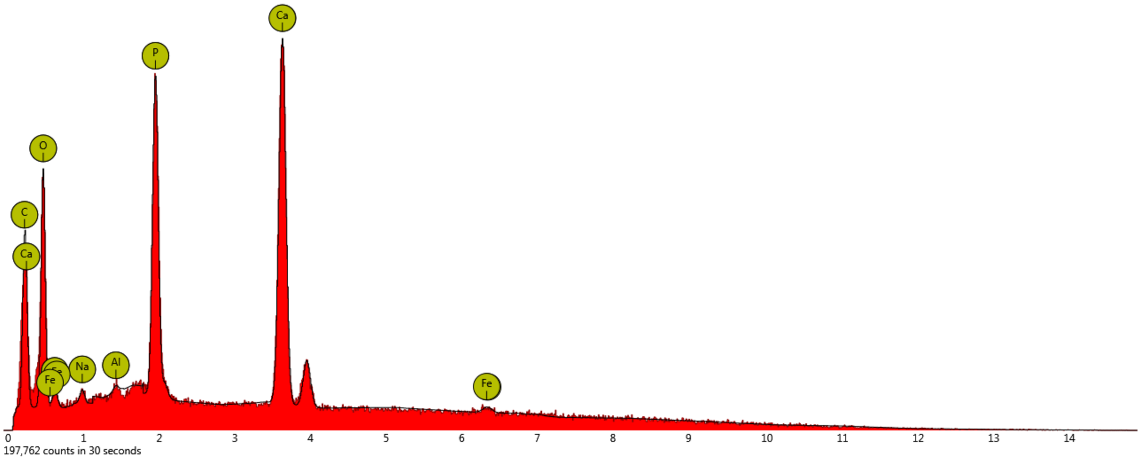

Disabled elements: N

Element Number	Element Symbol	Element Name	Atomic Concentration	Error
20	Ca	Calcium	12.8	0.0
15	P	Phosphorus	9.0	0.0
8	O	Oxygen	63.7	0.0
6	C	Carbon	7.6	2.1
9	F	Fluorine	4.8	1.6
11	Na	Sodium	1.5	0.8
26	Fe	Iron	0.4	1.5
13	Al	Aluminium	0.2	0.9

2. spot

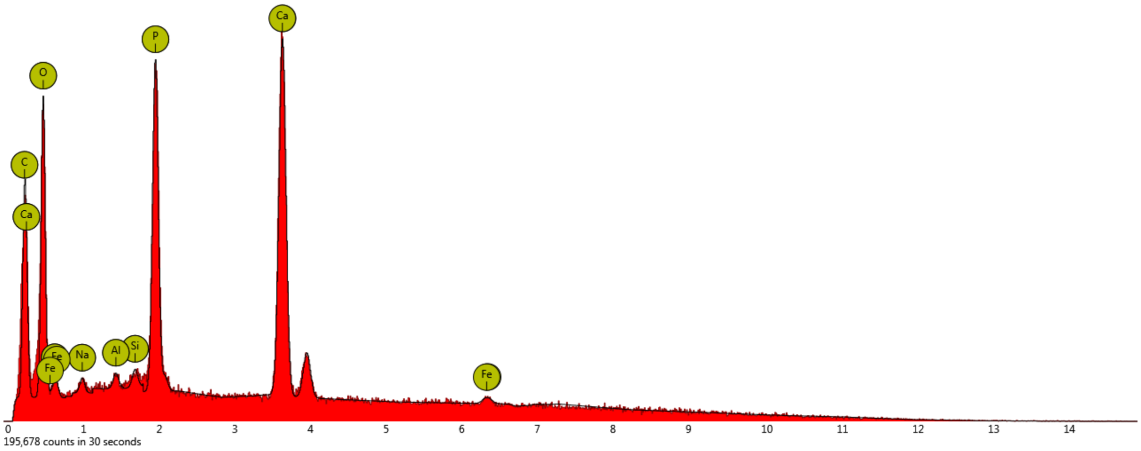

Disabled elements: N, Sr, Y

Element Number	Element Symbol	Element Name	Atomic Concentration	Error
20	Ca	Calcium	10.9	0.1
15	P	Phosphorus	8.2	0.1
8	O	Oxygen	64.8	0.0
6	C	Carbon	8.1	2.0
14	Si	Silicon	0.4	8.6
9	F	Fluorine	5.3	0.6
13	Al	Aluminium	0.5	0.3
11	Na	Sodium	1.4	1.6
26	Fe	Iron	0.5	0.5

3. spot

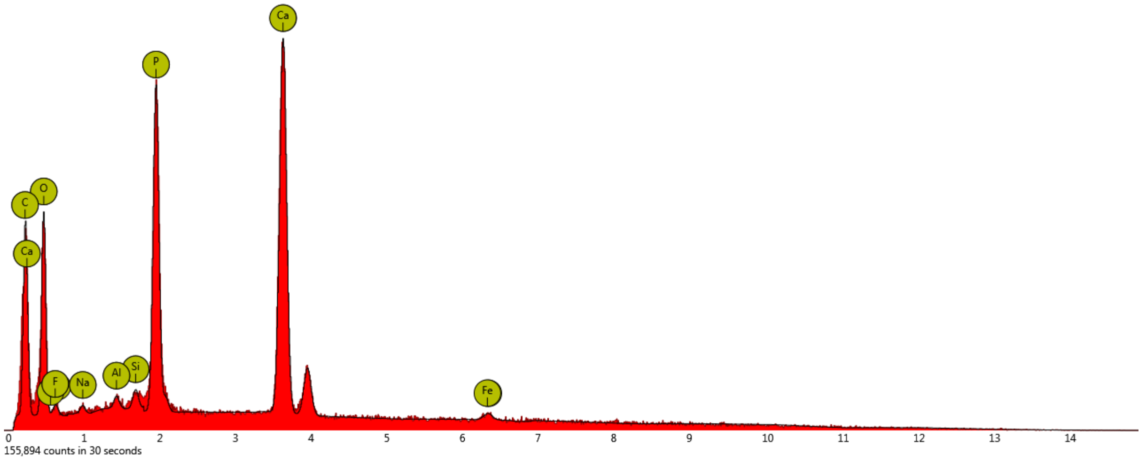

Disabled elements: B

Element Number	Element Symbol	Element Name	Atomic Concentration	Error
20	Ca	Calcium	13.9	0.0
15	P	Phosphorus	9.8	0.1
8	O	Oxygen	61.3	0.0
6	C	Carbon	9.2	1.9
14	Si	Silicon	0.5	16.4
13	Al	Aluminium	0.4	0.5
26	Fe	Iron	0.6	0.8
9	F	Fluorine	3.4	1.5
11	Na	Sodium	0.9	0.7

Bone matrix 01 (4 spots marked with cross and number)

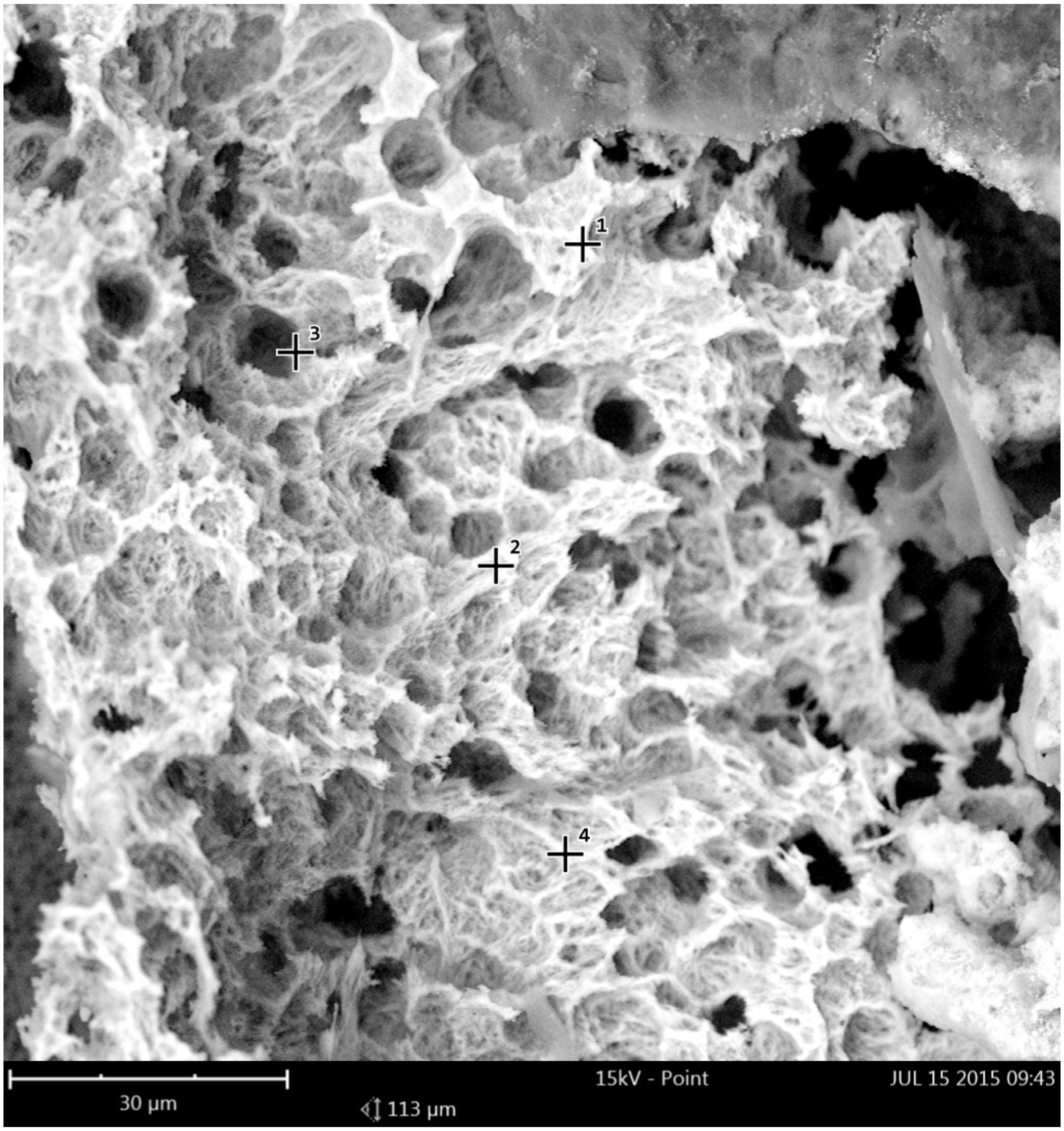

1. spot

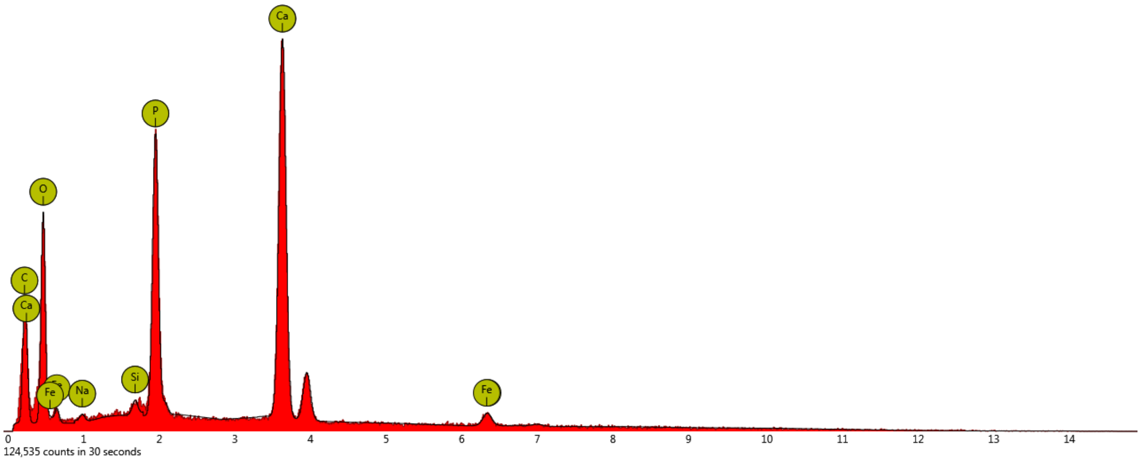

Disabled elements: N

Element Number	Element Symbol	Element Name	Atomic Concentration	Error
20	Ca	Calcium	15.0	0.0
15	P	Phosphorus	9.0	0.0
8	O	Oxygen	67.5	0.0
6	C	Carbon	6.1	1.9
26	Fe	Iron	1.2	0.2
14	Si	Silicon	0.4	5.2
11	Na	Sodium	0.8	2.6

2. spot

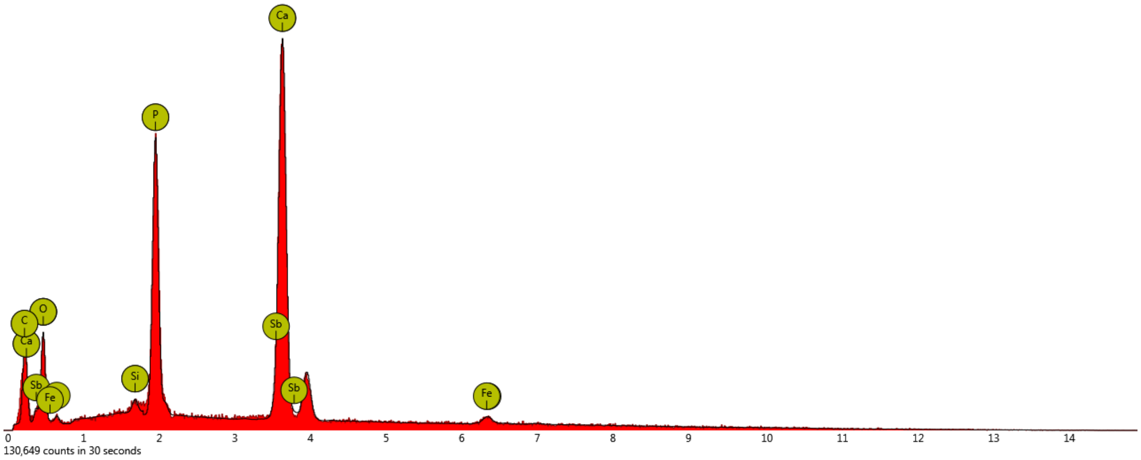

Disabled elements: B

Element Number	Element Symbol	Element Name	Atomic Concentration	Error
20	Ca	Calcium	23.3	0.0
15	P	Phosphorus	13.8	0.1
51	Sb	Antimony	0.9	0.0
8	O	Oxygen	53.7	0.0
6	C	Carbon	6.7	1.6
14	Si	Silicon	0.6	14.0
26	Fe	Iron	1.0	0.5

3. spot

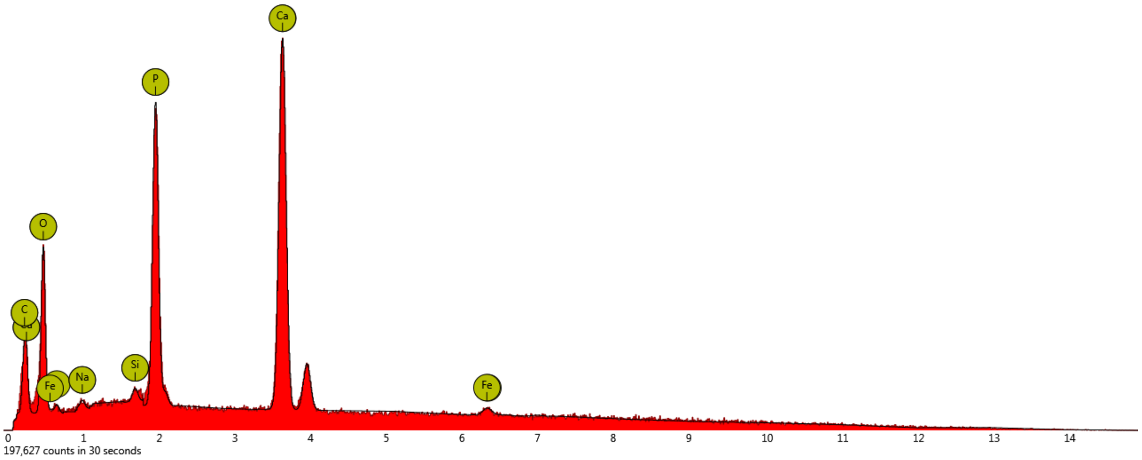

Disabled elements: N

Element Number	Element Symbol	Element Name	Atomic Concentration	Error
20	Ca	Calcium	16.9	0.1
15	P	Phosphorus	11.0	0.0
8	O	Oxygen	65.0	0.0
6	C	Carbon	4.9	1.4
14	Si	Silicon	0.4	4.0
26	Fe	Iron	0.7	0.7
11	Na	Sodium	1.1	1.5

4. spot

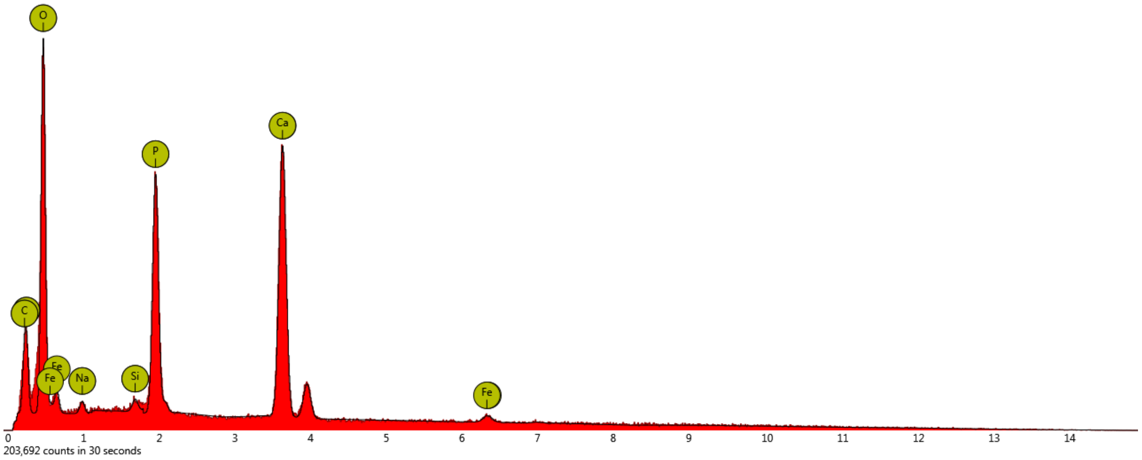

Disabled elements: N

Element Number	Element Symbol	Element Name	Atomic Concentration	Error
20	Ca	Calcium	9.3	0.0
8	O	Oxygen	78.5	0.0
15	P	Phosphorus	6.7	0.0
6	C	Carbon	3.4	1.6
14	Si	Silicon	0.3	7.2
11	Na	Sodium	1.3	0.6
26	Fe	Iron	0.5	0.5

Bone matrix 02 (2 spots marked with cross and number)

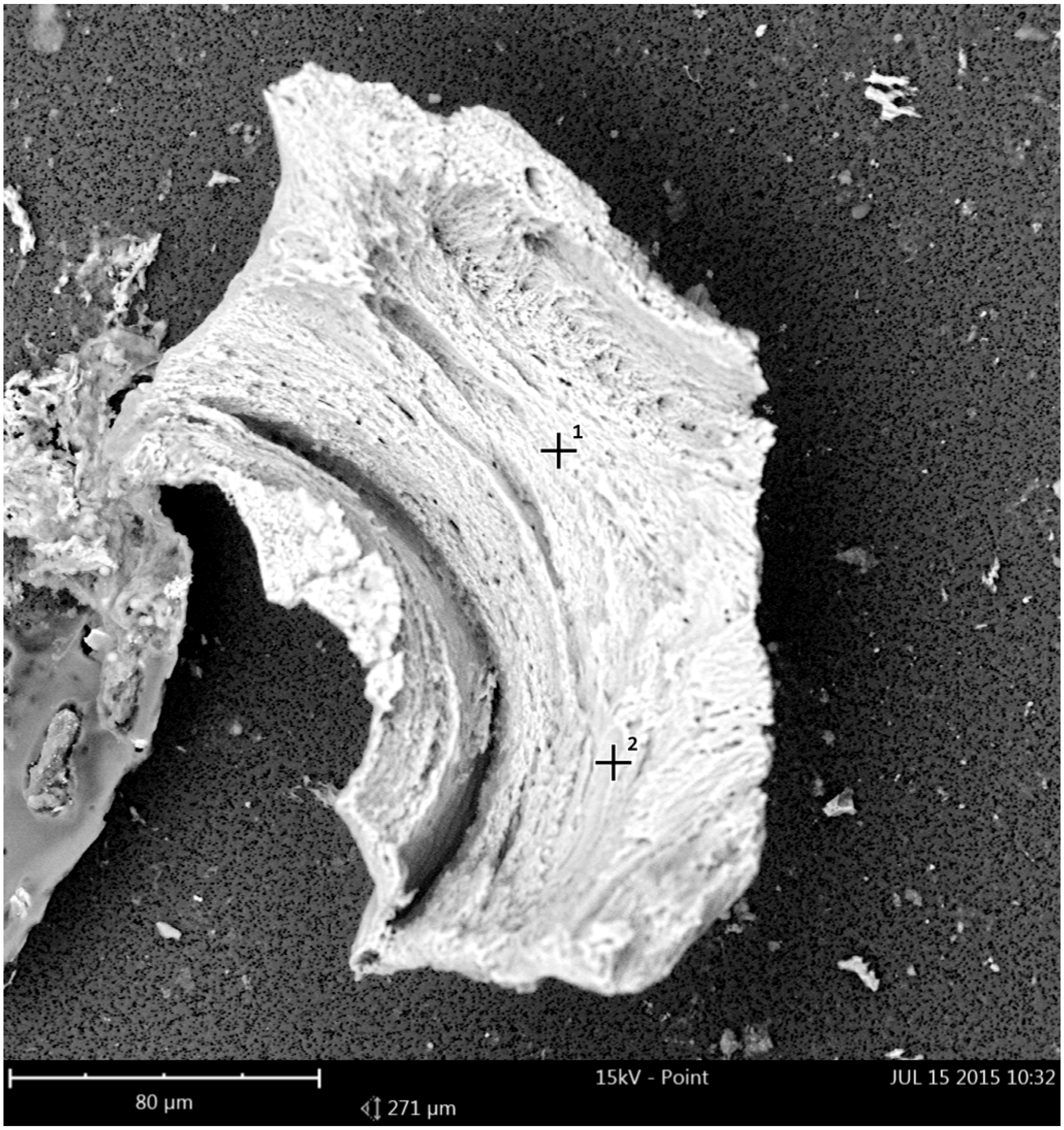

1. spot

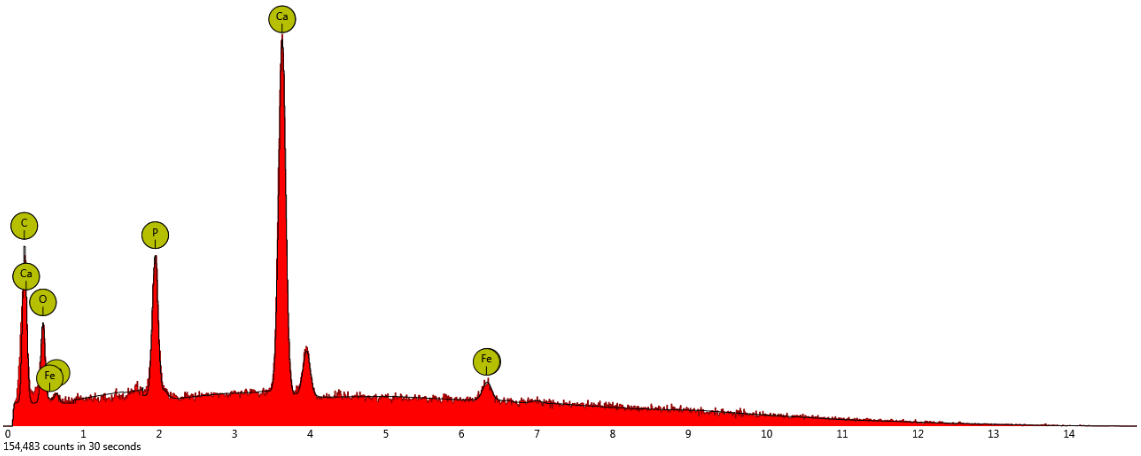

Disabled elements: B

**Element Number**	**Element Symbol**	**Element Name**	**Atomic Concentration**	**Error**
20	Ca	Calcium	23.4	0.0
15	P	Phosphorus	7.7	0.1
6	C	Carbon	11.6	1.1
8	O	Oxygen	54.4	0.1
26	Fe	Iron	2.9	0.6

2. spot

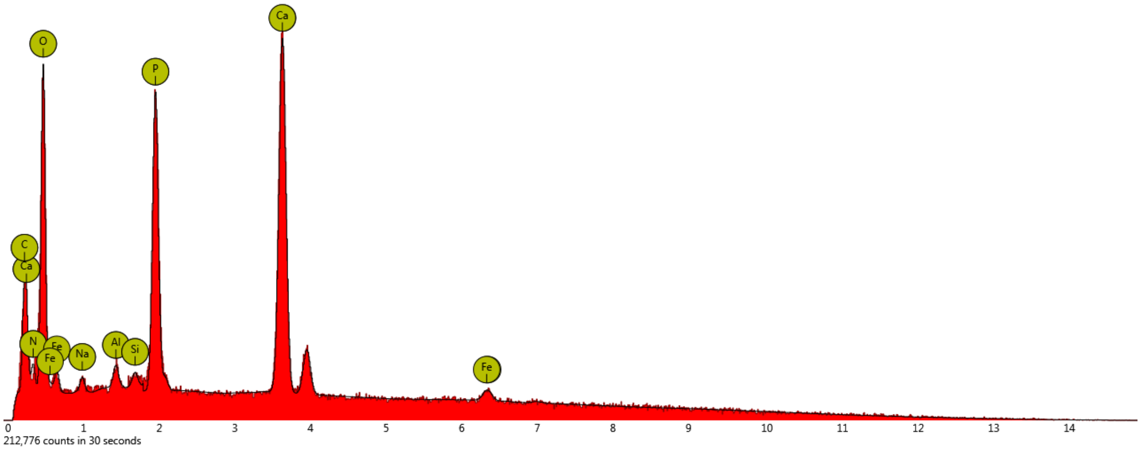

**Element Number**	**Element Symbol**	**Element Name**	**Atomic Concentration**	**Error**
20	Ca	Calcium	10.2	0.1
15	P	Phosphorus	7.0	0.0
8	O	Oxygen	69.0	0.0
6	C	Carbon	4.4	1.3
7	N	Nitrogen	6.4	2.0
13	Al	Aluminium	0.6	0.1
26	Fe	Iron	0.7	0.5
14	Si	Silicon	0.3	8.0
11	Na	Sodium	1.3	0.7
